# Baicalein Resensitizes Multidrug-Resistant Gram-Negative Pathogens to Doxycycline

**DOI:** 10.1128/spectrum.04702-22

**Published:** 2023-04-18

**Authors:** Yuhang Wang, Junfeng Su, Ziyan Zhou, Jie Yang, Wenjuan Liu, Yafen Zhang, Pengyu Zhang, Tingting Guo, Guocai Li

**Affiliations:** a Department of Microbiology, Institute of Translational Medicine, Medical College, Yangzhou University, Yangzhou, People’s Republic of China; b Jiangsu Key Laboratory of Experimental & Translational Non-coding RNA Research, Yangzhou, People’s Republic of China; c Jiangsu Key Laboratory of Zoonosis/Jiangsu Co-Innovation Center for Prevention and Control of Important Animal Infectious Diseases and Zoonoses, Yangzhou University, Yangzhou, People’s Republic of China; d Laboratory Department, Affiliated Hospital of Yangzhou University, Yangzhou, People’s Republic of China; JMI Laboratories

**Keywords:** baicalein, multidrug-resistant, Gram-negative pathogens, doxycycline

## Abstract

As multidrug-resistant pathogens emerge and spread rapidly, novel antibiotics urgently need to be discovered. With a dwindling antibiotic pipeline, antibiotic adjuvants might be used to revitalize existing antibiotics. In recent decades, traditional Chinese medicine has occupied an essential position in adjuvants of antibiotics. This study found that baicalein potentiates doxycycline against multidrug-resistant Gram-negative pathogens. Mechanism studies have shown that baicalein causes membrane disruption by attaching to phospholipids on the Gram-negative bacterial cytoplasmic membrane and lipopolysaccharides on the outer membrane. This process facilitates the entry of doxycycline into bacteria. Through collaborative strategies, baicalein can also increase the production of reactive oxygen species and inhibit the activities of multidrug efflux pumps and biofilm formation to potentiate antibiotic efficacy. Additionally, baicalein attenuates the lipopolysaccharide-induced inflammatory response *in vitro*. Finally, baicalein can significantly improve doxycycline efficacy in mouse lung infection models. The present study showed that baicalein might be considered a lead compound, and it should be further optimized and developed as an adjuvant that helps combat antibiotic resistance.

**IMPORTANCE** Doxycycline is an important broad-spectrum tetracycline antibiotic used for treating multiple human infections, but its resistance rates are recently rising globally. Thus, new agents capable of boosting the effectiveness of doxycycline need to be discovered. In this study, it was found that baicalein potentiates doxycycline against multidrug-resistant Gram-negative pathogens *in vitro* and *in vivo*. Due to its low cytotoxicity and resistance, the combination of baicalein and doxycycline provides a valuable clinical reference for selecting more effective therapeutic strategies for treating infections caused by multidrug-resistant Gram-negative clinical isolates.

## INTRODUCTION

Antibiotics are an important part of modern medicine and constitute the primary method of treating bacterial infections. However, the development of multidrug resistance (MDR) in patients complicates antimicrobial therapy, particularly for Gram-negative pathogens that have highly impermeable outer membranes; thus, MDR causes a clinical dilemma for clinicians (1). Globally, carbapenem-resistant Gram-negative opportunistic pathogens are rated as the top priority, along with Pseudomonas aeruginosa, Klebsiella pneumoniae, and Acinetobacter baumannii (2).

Gram-negative opportunistic pathogens develop various defense mechanisms for resisting the hazardous effect of antibiotics. The limited efficacy of antibiotics is due to the presence of MDR genes and intrinsic tolerance mechanisms, such as drug efflux (3), biofilm formation (4), a unique impermeable outer membrane barrier (5), and other phenotypic resistance mechanisms. This phenotypic resistance is often associated with the tricarboxylic acid (TCA) cycle (6), proton motive force (PMF) (7), bacterial respiration, and reactive oxygen species (ROS) (8). For mitigating antibiotic resistance, a suitable alternative to the search for new antibiotics is the development of innovative strategies to increase the potency of known antibiotics.

Doxycycline is a broad-spectrum antibiotic targeting bacterial ribosomes that belongs to the tetracycline class. It is extensively used in clinics (9). However, with the indiscriminate use of antibiotics, the prevalence of doxycycline-resistant pathogens threatens public safety (10). The *tetA* efflux pump family is closely associated with doxycycline resistance (11). Therefore, developing practical approaches to repress *tetA* and combat doxycycline resistance is essential. Several studies have shown that traditional Chinese medicine combined with antibiotics is effective against bacterial infections (12–14).

Baicalein is a bioactive flavonoid extracted from the roots of Scutellaria baicalensis Georgi. It has been used to treat bacterial infections since ancient times (15). Jang et al. reported that baicalein inhibits *tetK* and prevents tetracycline from effluxing, thus resensitizing Escherichia coli to tetracycline (16). The methicillin-resistant Staphylococcus aureus strains OM481 and OM584 were suppressed synergistically by baicalein and tetracycline (17). Baicalein also has a synergistic effect when combined with cefotaxime on certain K. pneumoniae strains expressing *CTX-M-1* mRNA (18). Baicalein with gentamicin has a synergistic effect on vancomycin-resistant *Enterococcus* isolates (19). However, the mechanism through which baicalein acts as a broad-spectrum antibacterial adjuvant is not fully known.

In this study, we analyzed the broad-spectrum antibacterial adjuvant baicalein and the synergistic effect of baicalein combined with doxycycline. Detailed studies showed that baicalein could attach to phospholipids, compete with Mg^2+^ to bind to lipid A, and exert antimicrobial effects on Gram-negative bacteria. Additionally, baicalein accelerated the TCA cycle, enhanced oxidative damage and bacterial cell membrane potential (ΔΨ) depolarization, leading to inhibition of multidrug efflux systems, and biofilm formation, thus improving the efficacy of doxycycline. Baicalein also down-modulated the lipopolysaccharide-induced host inflammatory response. These findings suggested that baicalein combined with doxycycline is efficacious in treating disorders resulting from MDR Gram-negative pathogens. Our study provides novel insights into treatment strategies to combat such infections.

## RESULTS

### Baicalein is a potent broad-spectrum antibiotic adjuvant.

**FIG 1:**
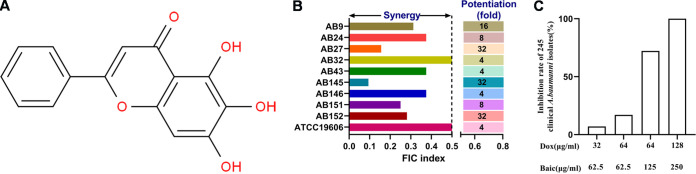
Baicalein combined with doxycycline was effective against A. baumannii. (A) Chemical structure of baicalein. (B) Using a checkerboard microdilution assay, the synergy between baicalein and doxycycline against 10 representative clinical isolates of A. baumannii was determined. The degree of doxycycline potentiation in the presence of 0.25× MIC of baicalein was evaluated. Synergy was defined as a FICI of ≤0.5. (C) Combinations of baicalein and doxycycline against 245 clinical isolates of A. baumannii. According to the CLSI 2021 guidelines, when bacteria coincubated with baicalein and doxycycline for 18 h at 37°C exhibited no visible growth, we considered that growth inhibition occurred. Dox, doxycycline; Baic, baicalein. The data are based on three biological replicates.

**FIG 2:**
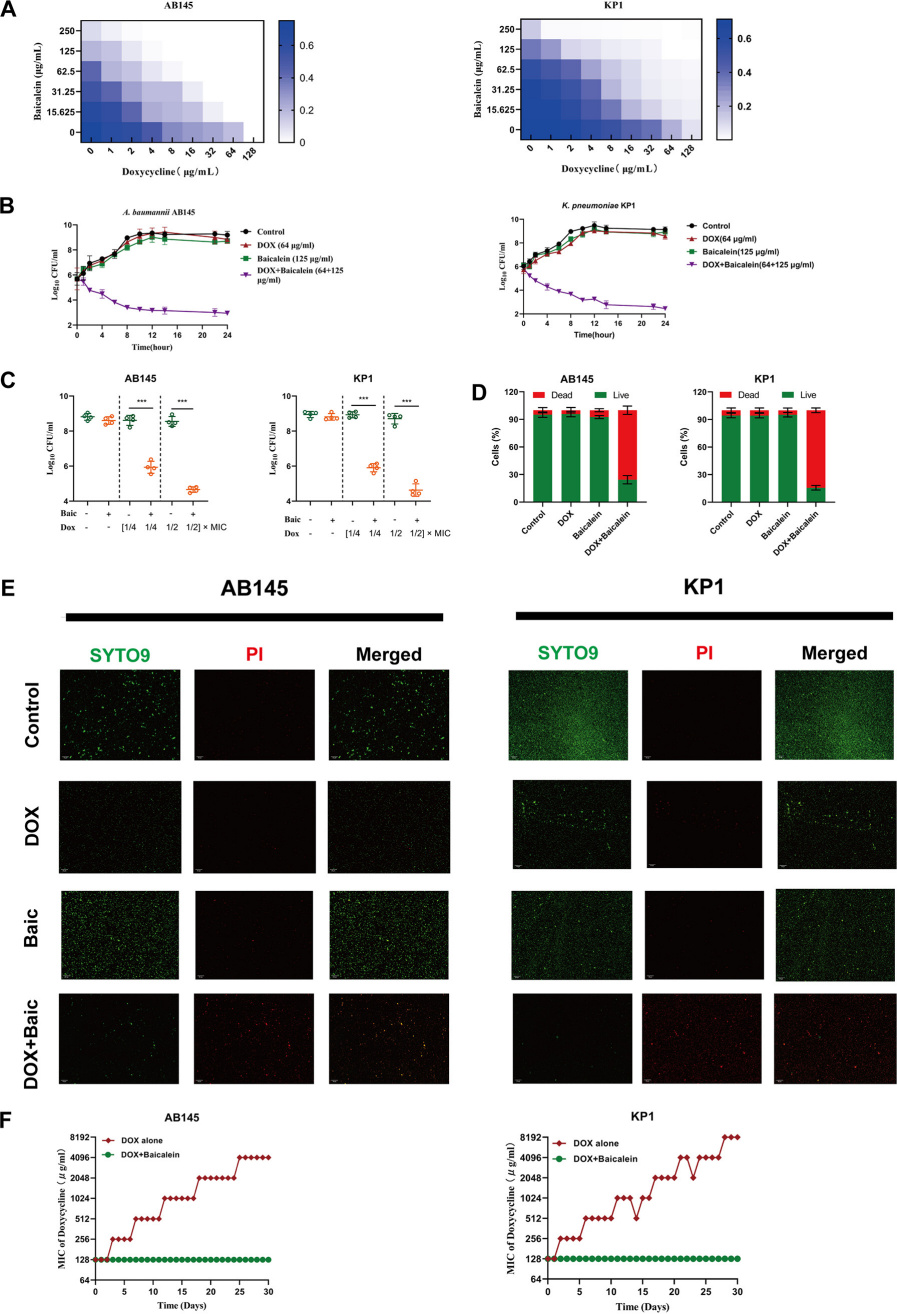
Baicalein synergy with doxycycline has antibacterial activity against *tetA*-positive Gram-negative pathogens *in vitro*. (A) Synergistic antimicrobial effects of baicalein and doxycycline on A. baumannii AB145 and K. pneumoniae KP1, determined by the checkerboard assay. Darker blue areas indicate higher cell density and lower inhibition caused by combinatorial therapy. (B) Time-dependent killing curve of A. baumannii AB145 and K. pneumoniae KP1 with a combinatorial treatment of baicalein and doxycycline. Pathogens were grown to the exponential phase in MHB and then were treated with PBS, a sub-MIC of doxycycline (DOX, 64 μg/mL), a sub-MIC of baicalein (125 μg/mL), or both (DOX+baicalein; 64 μg/mL + 125 μg/mL) for 24 h. (C) Viable counts of bacteria after treatment with baicalein (62.5 μg/mL) alone or in combination with doxycycline (1/4× MIC or 1/2× MIC) for 16 h. The data are mean ± SD, and differences were assessed using nonparametric one-way ANOVA; ***, *P < *0.001. (D) The LIVE/DEAD BacLight bacterial viability kit was used to evaluate the percentage of live and dead bacteria. (E) Representative fluorescent images of A. baumannii AB145 and K. pneumoniae KP1 live (green fluorescence) and dead (red fluorescence) bacterial cells after treatment with baicalein (62.5 μg/mL) and doxycycline (32 μg/mL) alone or in combination for 8 h. Bar, 50 μm. (F) Adding baicalein (62.5 μg/mL, 1/4× MIC) prevented doxycycline resistance in A. baumannii AB145 and K. pneumoniae KP1 *in vitro*. Resistance was acquired during serial passage in 0.25× MIC of doxycycline. The data are based on three biological replicates and presented as the mean ± SD.

For our previously constructed extensively drug-resistant (XDR) strain A. baumannii AB43Δ*crispr-cas* (20), a checkerboard assay showed that baicalein can act as a synergistic antibacterial activity (fractional inhibitory concentration index [FICI] ≤ 0.5) with most conventional antibiotics (10/14, 71.43%), including doxycycline (FICI = 0.1875) (Table 1). A similar pattern was found for the sensitive strain AB43 (Table S1). The combination of baicalein and doxycycline was effective against 245 clinical isolates of A. baumannii along with other *tetA*-positive MDR and pandrug-resistant (PDR) strains of Gram-negative pathogens (Fig. 1; Table 2). Our time-dependent killing curve showed that the combination treatment had good antibacterial activity against the *tetA*-positive MDR strains A. baumannii AB145 (FICI = 0.09375) and K. pneumoniae KP1 (FICI = 0.1875) (Fig. 2A and B). Relative to doxycycline alone, 62.5 μg/mL baicalein significantly enhanced the antimicrobial activity of doxycycline and reduced the number of viable AB145 and KP1 bacteria after 16 h of treatment *in vitro* (Fig. 2C). The results of the live/dead bacterial staining assay showed that the cell survival rate in the combined drug group was lower than that in the single-agent group (Fig. 2D and E).

**TABLE 1:** Synergistic activity of baicalein in combination with different classes of antibiotics against extensively drug-resistant AB43Δ*crispr-cas*

Antibiotic	MIC (μg/mL)	FICI^*a*^	MIC with baicalein (μg/mL)^*b*^	Potentiation (fold)
Ampicillin	10,192	0.28125	637	16
Doxycycline	128	0.1875	8	16
Erythromycin	32	0.375	2	16
Rifampin	8	0.25	1	8
Minocycline	4	0.3125	0.0625	64
Tetracycline	2,048	0.375	512	4
Tigecycline	64	0.375	8	8
Imipenem	16	0.25	4	4
Gentamicin	256	0.5	64	4
Cefoperazone and sulbactam	128	0.25	16	8
Ceftriaxone sodium	256	0.75	128	2
Ciprofloxacin	16	>1	≥16	≤1
Oxytetracycline	32	1	16	2
Vancomycin	32	1.5	32	1

a FICIs were calculated based on checkerboard broth microdilution assays. Effects are defined as follows: synergy, FICI ≤ 0.5; additive, 0.5 < FICI < 1; indifferent, 1 ≤ FICI < 4.

b MIC in the presence of 62.5 μg/mL of baicalein.

**TABLE 2:** Baicalein enhances the antimicrobial activity of doxycycline against Gram-negative pathogens

Strain	Genotype^*a*^	Resistance pattern	Doxycycline		Baicalein		FICI^*b*^
			MIC (μg/mL)	FIC	MIC (μg/mL)	FIC	
A. baumannii							
AB9	*tetA*^+^ *tetB*^+^	MDR	128	0.0625	250	0.25	0.3125
AB24	*tetB* ^+^	PDR	128	0.125	500	0.25	0.375
AB27	*tetA*^+^ *tetB^+^*	MDR	128	0.03125	250	0.125	0.15625
AB32	*tetA*^+^ *tetB^+^*	PDR	128	0.25	250	0.25	0.5
AB43	*tetA tetB*	Sensitive	1	0.25	250	0.125	0.375
AB145	*tetA* ^+^	MDR	128	0.03125	250	0.0625	0.09375
AB146	*tetB^+^*	PDR	128	0.25	250	0.125	0.375
AB151	*tetA tetB*	PDR	64	0.125	500	0.125	0.25
AB152	*tetA*^+^ *tetB^+^*	MDR	64	0.03125	500	0.25	0.28125
ATCC 19606	*tetA tetB*	Type strain, non-MDR	1	0.25	250	0.25	0.5
K. pneumoniae							
KP1	*tetA* ^+^	MDR	128	0.125	250	0.0625	0.1875
KP2	*tetA* ^+^	PDR	128	0.25	500	0.125	0.375
KP6	*tetA* ^+^	PDR	64	0.125	500	0.25	0.375
KP10	*tetA* ^+^	MDR	64	0.0625	125	0.125	0.1875
KP12	*tetA* ^+^	MDR	64	0.03125	250	0.25	0.28125
KP13	*tetA* ^+^	PDR	64	0.0625	500	0.25	0.3125
KP14	*tetA* ^+^	PDR	32	0.125	500	0.25	0.375
KP15	*tetA* ^+^	MDR	32	0.125	250	0.125	0.25
E. coli							
*E.col*-1	*tetA* ^+^	MDR	32	0.125	250	0.25	0.375
*E.col*-2	*tetA* ^+^	MDR	64	0.0625	500	0.0625	0.125
*E.col*-3	*tetA* ^+^	MDR	32	0.125	250	0.125	0.25
P. aeruginosa							
PA-1	*tetA* ^+^	MDR	64	0.125	250	0.125	0.25
PA-2	*tetA* ^+^	MDR	128	0.125	500	0.125	0.25

a +, presence; no symbol, absence.

b The FICI is the sum of the FICs of doxycycline and baicalein. All effects shown here are defined as synergistic (FICI ≤ 0.5).

Whether the combination therapy can exacerbate single-agent toxicities needs to be determined. Further examination of the biological safety of this synergism showed that even at high concentrations, the combination of baicalein and doxycycline caused only 1% hemolysis of sheep red blood cells (see Fig. S1A in the supplemental material). The mammalian cell cytotoxicity results consistently showed that the synergism of baicalein and doxycycline had low cytotoxicity (Fig. S1B to D) (21). Antibiotic resistance is a significant challenge in the treatment of bacterial infections. Serial passaging in the presence of doxycycline for 31 days increased MICs for A. baumannii AB145 and K. pneumoniae KP1 by 32- and 64-fold, respectively, whereas the combination treatment prevented the development of resistance (Fig. 2F). It is worth noting that the per-generation doxycycline-resistant mutants exhibited cross-resistance (22) to different classes of antibiotics, and the expression levels of *tetA* increased significantly in some high-level doxycycline-resistant mutants (Fig. S2; Table S2). Moreover, all doxycycline-resistant mutants contained multiple nonsynonymous mutations in the genes of *tetA* (Table S3). These results indicated that baicalein could inhibit doxycycline resistance in *tetA*-positive MDR A. baumannii and K. pneumoniae KP1.

### Baicalein disrupts the inner and outer membranes of Gram-negative bacteria.

**FIG 3:**
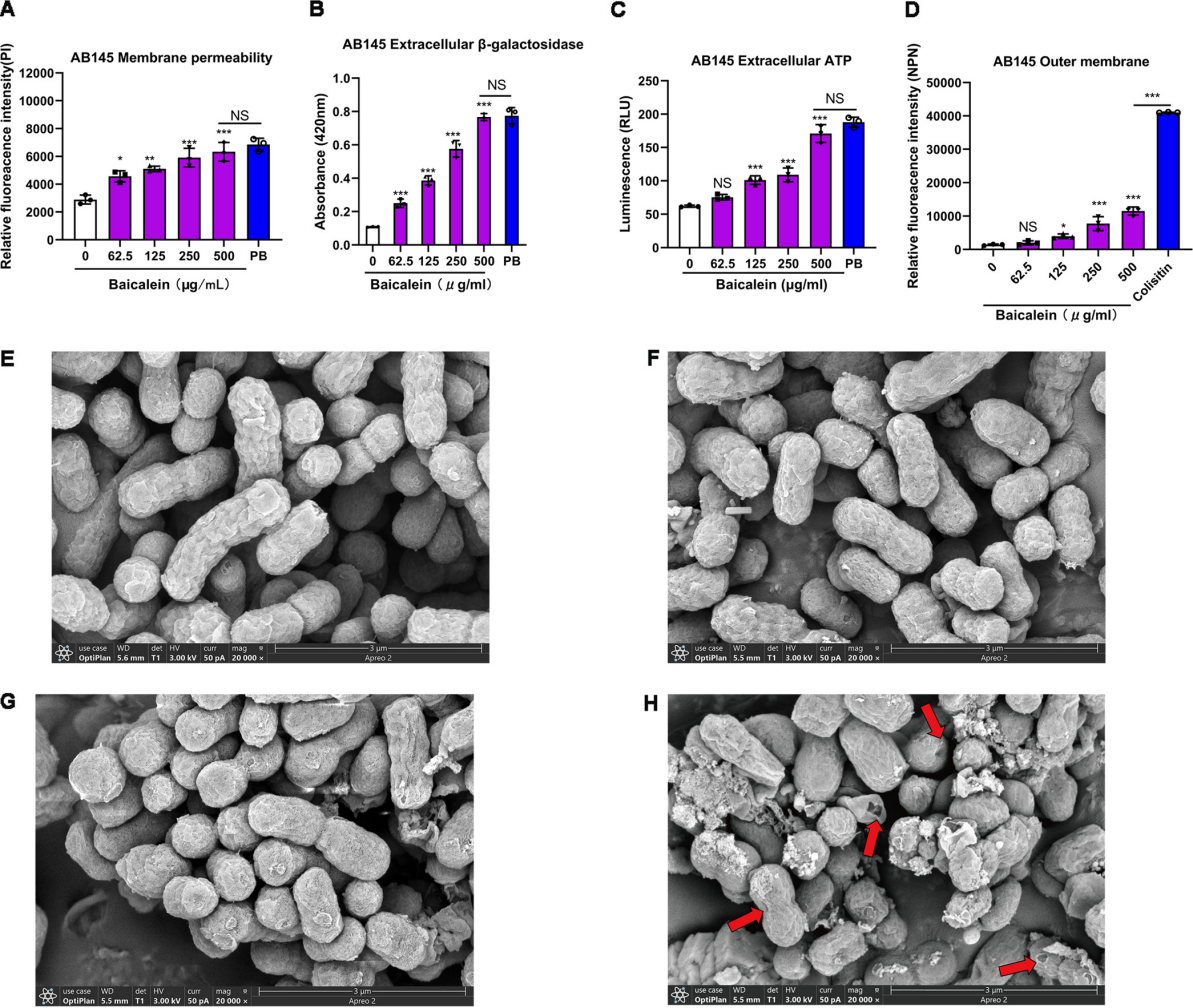
Baicalein exerts antibacterial activity through the inner and outer membranes of A. baumannii. (A) The permeability of the cytoplasmic membrane of A. baumannii AB145 increased after treatment with baicalein (0 to 500 μg/mL) or PB (16 μg/mL) for 30 min and probing with 10 nmol/L of PI. (B and C) The release of bacterial contents, including extracellular (B) β-galactosidase (molecular weight [MW], 130 kDa) and (C) ATP (MW, 507 Da), increased after treatment with baicalein (0 to 500 μg/mL) for 30 min. (D) The permeability of the outer membrane of A. baumannii AB145 increased after treatment with baicalein (0 to 500 μg/mL) or colistin (4 μg/mL) for 30 min and probing with 10 μmol/L of NPN. The data in panels A to D represent three biological replicates, and the error bars represent SD. The *P* values were determined by performing nonparametric one-way ANOVA. NS, not significant; *, *P < *0.05; **, *P < *0.01; ***, *P < *0.001. (E to H) Morphological changes in A. baumannii AB145 after 1-h treatment with PBS (E), a sub-MIC of doxycycline (64 μg/mL) (F), a sub-MIC of baicalein (125 μg/mL) (G), or the combination (H), visualized using SEM. Bar = 3 μm. Red arrows indicate the destroyed outer membrane.

Baicalein showed synergistic effects with multiple antibiotics against Gram-negative pathogens. We inferred that baicalein affects the endogenous mechanism of resistance to antibiotics in Gram-negative bacteria. The cell envelope of Gram-negative bacteria is the first line of defense against antibiotics (23). In this study, the fluorescence intensity of propidium iodide (PI) and 1-*N*-phenylnaphthylamine (NPN) and the extracellular content of β-galactosidases and ATP increased with the concentration of baicalein (Fig. 3 and Fig. S3). The scanning electron microscopy (SEM) images revealed that after the combination treatment, the cell surface was depressed and the cell shrank, collapsed, and underwent lysis, but these changes did not occur in untreated cells. This indicated that the effect of baicalein on bacteria might be associated with the rapidly disrupted cell wall and the inner and outer membranes of the cell.

**FIG 4:**
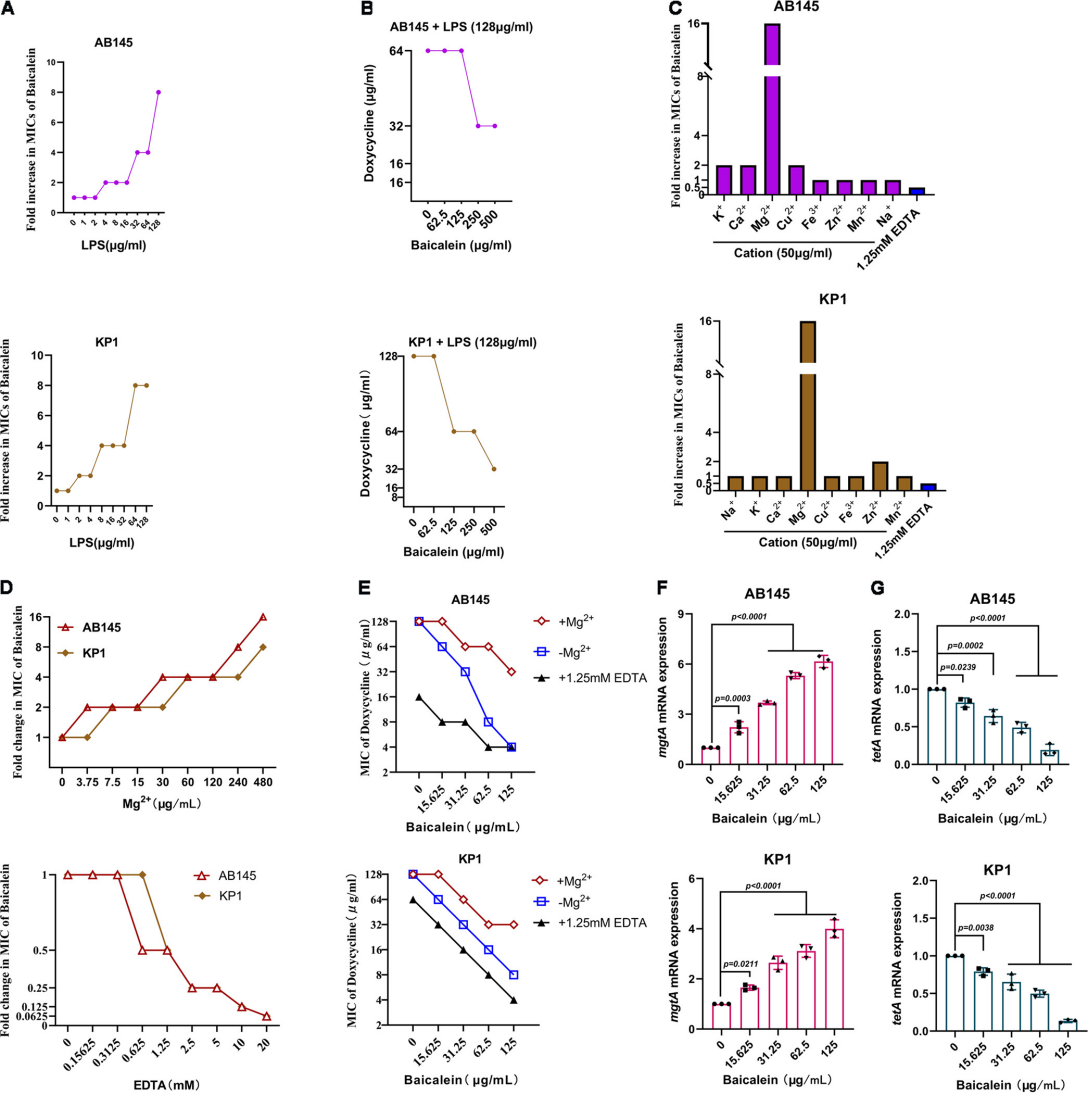
Baicalein competes with Mg^2+^ to bind to lipid A. (A) Exogenous addition of A. baumannii-derived or K. pneumoniae-derived LPS (0 to 128 μg/mL) impaired the antibacterial activity of baicalein against AB145 and KP1 in a dose-dependent manner, determined by a checkerboard microdilution assay. (B) Purified LPS (128 μg/mL) reduced the antibacterial activity of baicalein combined with doxycycline. (C) The effects of different cations (50 μg/mL) and EDTA (1.25 mM) on the antibacterial activity of baicalein against AB145 and KP1. (D) Mg^2+^ inhibited the antibacterial activity of baicalein against AB145 and KP1 in a dose-dependent manner; EDTA was used as a positive control. (E) The exogenous addition of Mg^2+^ (480 μg/mL) reduced the antibacterial activity of baicalein combined with doxycycline. (F) The expression of the *mgtA* mRNA, which belongs to the PhoPQ two-component system, presents increased with increasing baicalein does. (G) As determined by RT-PCR analysis, baicalein inhibited the transcription of *tetA* in a dose-dependent manner. The data are means ± SD, and the differences were assessed using nonparametric one-way ANOVA.

**FIG 5:**
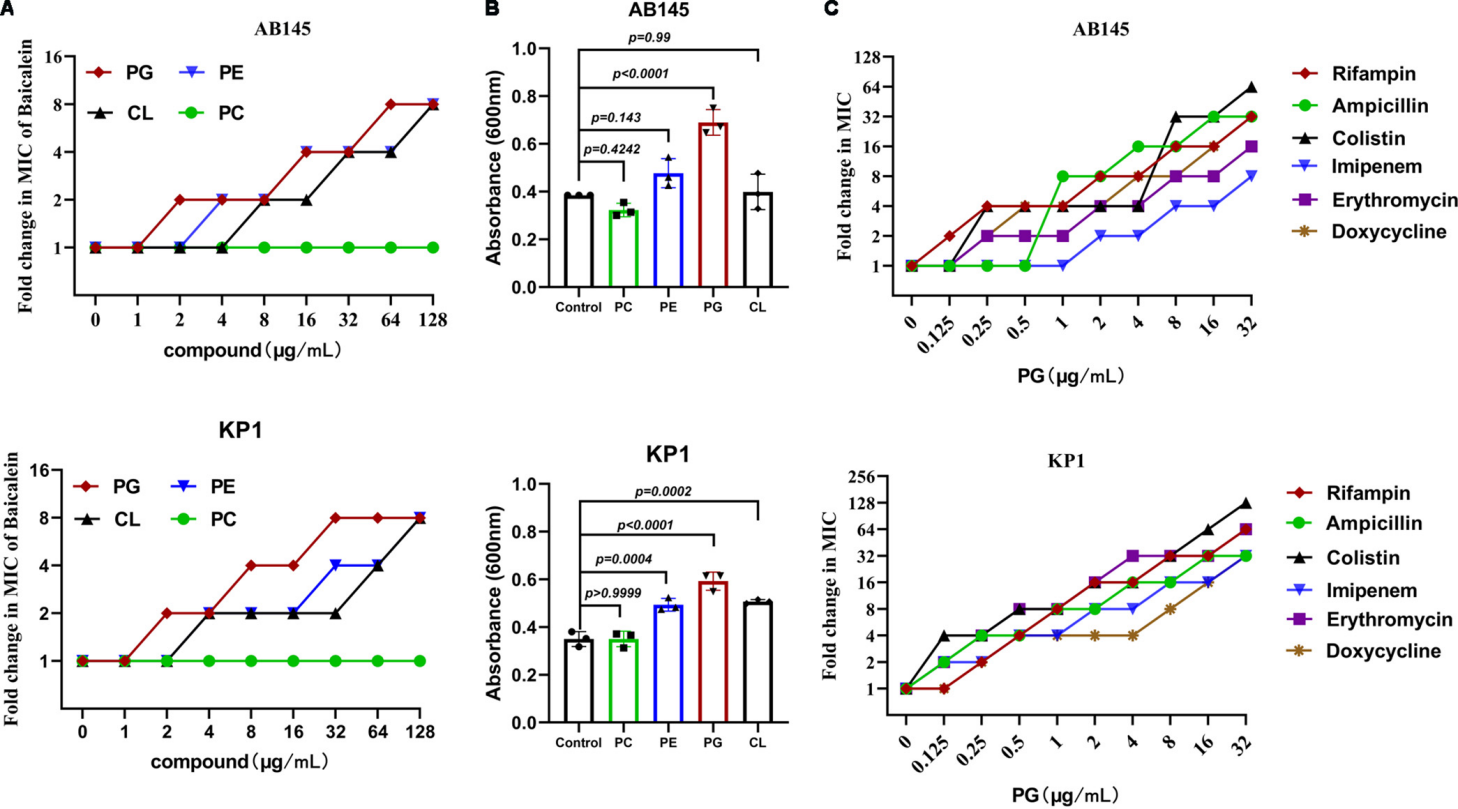
Phospholipids reduce the antibacterial activity of baicalein against A. baumannii and K. pneumoniae. (A) The MICs of baicalein against AB145 and KP1 increased in the presence of phospholipids (0 to 128 μg/mL), as determined by a checkerboard microdilution assay. PC was used as a control. (B) PG decreased the antibacterial activity of baicalein against AB145 and KP1. Turbidity of the mixtures of baicalein (sub-MIC, 50 μL) with various phospholipids (5 mg/mL, 50 μL) at 600 nm. (C) PG blocked the antibacterial activity of baicalein combined with multiple antibiotics (rifampin, ampicillin, colistin, imipenem, erythromycin, and doxycycline) against AB145 and KP1. A sub-MIC of baicalein was added to the bacterial suspension in the presence of PG (0 to 32 μg/mL) combined with each antibiotic. The data are means ± SD, and the differences were determined by nonparametric one-way ANOVA.

In Gram-negative bacteria, lipopolysaccharide (LPS) is a significant component of the outer membrane. The exogenous addition of LPS from the corresponding bacterial strain reduced the antibacterial activity of baicalin in a dose-dependent manner, and high concentrations of LPS (128 μg/mL) reduced the antibacterial activity of baicalein combined with doxycycline (Fig. 4A and B). Through ionic bridges, divalent cations can connect to the negatively charged phosphate groups among lipid A molecules, which along with core polysaccharides can form LPS. We found that the exogenous addition of K^+^, Ca^2+^, Mg^2+^, Cu^2+^, and Zn^2+^ inhibited the activity of baicalein, and Mg^2+^ strongly inhibited the antibacterial activity of baicalein combined with doxycycline, while Fe^3+^, Mn^2+^, and Na^+^ had no significant impact on these activities (Fig. 4C to E). As expected, the expression of *mgtA*, a transcriptional regulator responsible for Mg^2+^ starvation, showed a dose-dependent increase with baicalein treatment, but the opposite occurred with *tetA* (Fig. 4F and G). The main functional and structural components of the inner membrane of Gram-negative bacteria are phospholipids, which include phosphatidylethanolamine (PE), cardiolipin (CL), and phosphatidylglycerol (PG) (24). The MICs of baicalein were higher with the exogenous addition of phospholipids, and PG blocked the antibacterial activity of baicalein combined with antibiotics (Fig. 5). These findings suggested that baicalein-mediated membrane perturbation strongly promotes the antibacterial activity of doxycycline.

### Transcriptomic analysis of the antibacterial activity of baicalein combined with doxycycline.

**FIG 6:**
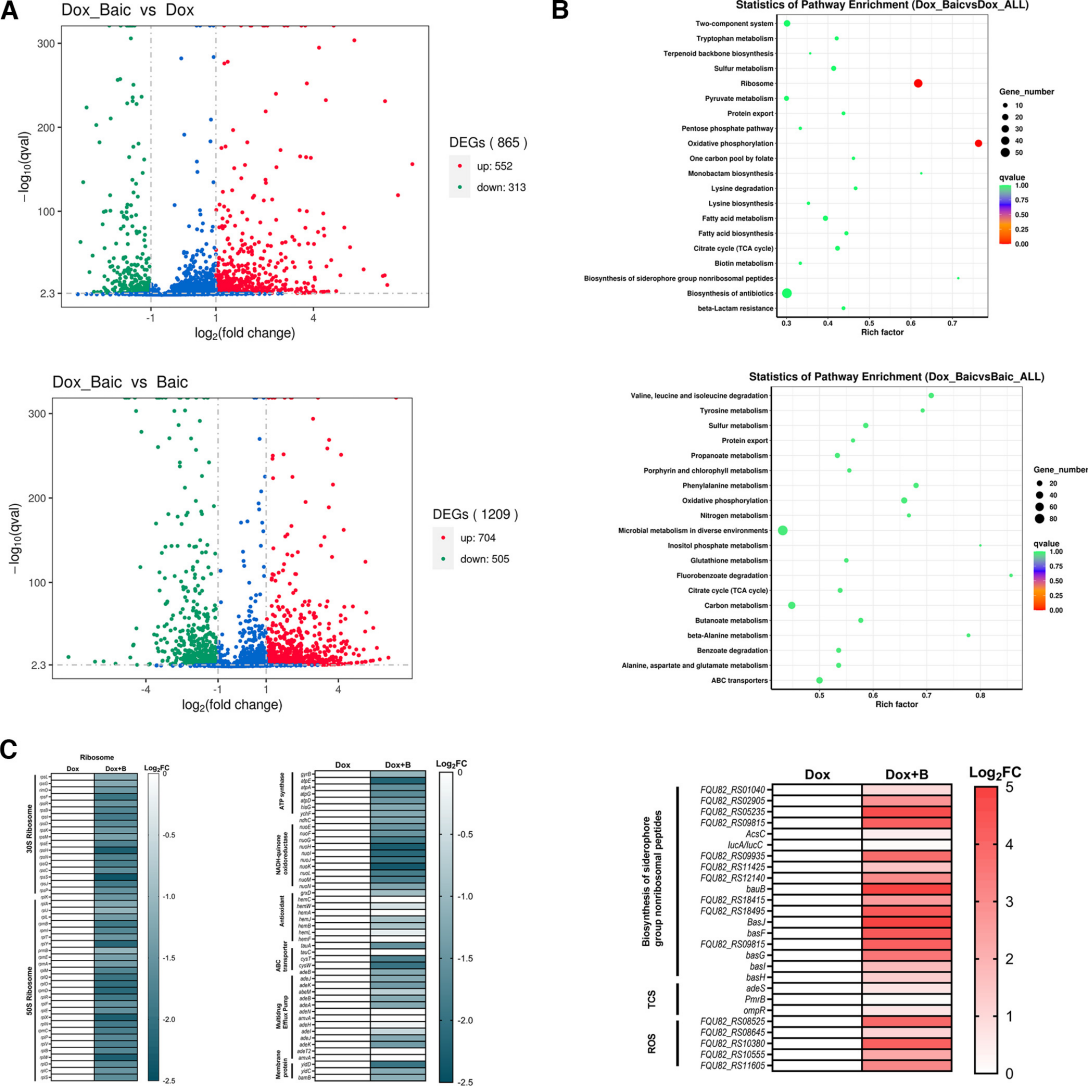
Transcriptomic analysis of A. baumannii AB145 after exposure to different treatments. Volcano plots illustrate the RNA sequencing transcriptome (A) and KEGG enrichment analysis (B) of the DEGs in AB145 after treatment with doxycycline (64 μg/mL), baicalein (125 μg/mL), or their combination for 4 h. In panel A, the *x* and *y* axes indicate the changes in the expression and the corresponding degree of statistical significance. (C) The selected DEGs were associated with ribosomes, oxidative phosphorylation, antioxidants, multidrug efflux pumps, ABC transporters, membrane proteins, the biosynthesis of siderophore group nonribosomal peptides, ROS, and the two-component system (TCS). DOX, doxycycline alone; DOX+B, combination of doxycycline and baicalein. The data are from three biological replicates.

To better understand the inhibitory effect of baicalein combined with doxycycline in Gram-negative bacteria, transcription analysis of A. baumannii AB145 was conducted following the administration of doxycycline, baicalein, or their combination for 4 h. Compared to the treatment with doxycycline alone, the combination treatment upregulated 552 genes and downregulated 313 genes, while the combination group showed 704 upregulated genes and 505 downregulated genes compared to the group treated with baicalein alone (Fig. 6A). The results of KEGG analysis showed that the common differentially expressed genes were associated with microbial metabolism, protein export, oxidative phosphorylation, TCA cycle, and ABC transporters (Fig. 6B). Additionally, genes related to ATP synthase, NADH-quinone oxidoreductase, the multidrug efflux pump, ABC transporters, and membrane proteins were downregulated, whereas ROS-related genes were significantly upregulated after combination treatment compared to after treatment with doxycycline alone (Fig. 6C). Thus, the antibacterial activity of baicalein combined with doxycycline might be associated with multiple pathways.

### Mechanisms by which baicalein potentiates doxycycline activity.

**FIG 7:**
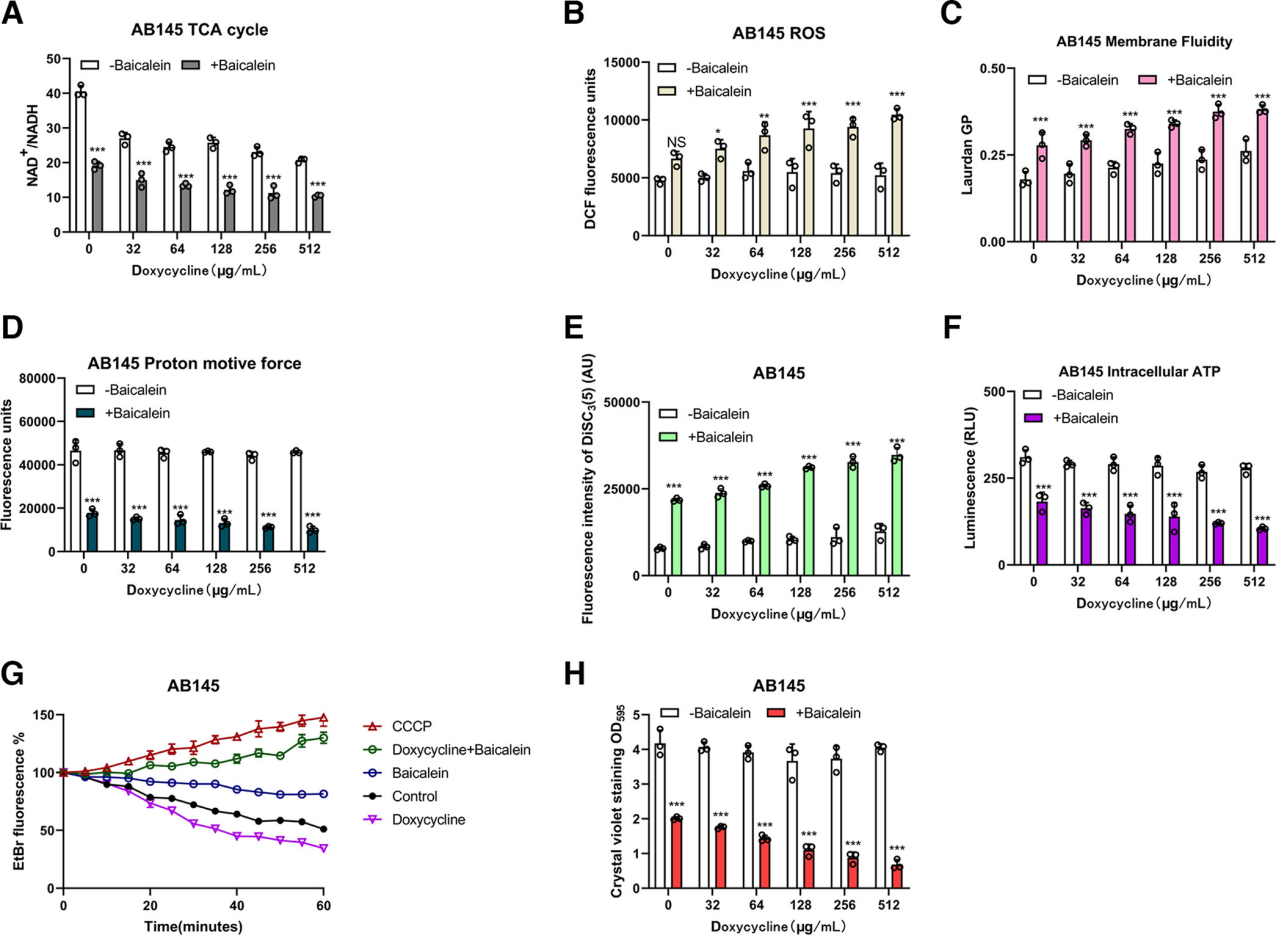
Synergistic mechanisms of baicalein combined with doxycycline against A. baumannii. (A) Combining baicalein (125 μg/mL) and doxycycline enhanced the activity of the TCA cycle. (B) Adding baicalein (125 μg/mL) to doxycycline increased ROS generation. (C) Membrane fluidity of AB145 decreased after combined treatment. (D) Baicalein (125 μg/mL) combined with doxycycline promoted PMF dissipation, assessed by monitoring the fluorescence intensity of BCECF-AM-probed AB145 cells. (E) The combined treatment promoted bacterial ΔΨ depolarization, evaluated by measuring the fluorescence intensity of DiSC_3_(5). (F) Decrease in the intracellular ATP levels in AB145 after the colocalization of baicalein with doxycycline. (G) Inhibition of EtBr efflux pumps by treatment with baicalein (125 μg/mL) combined with doxycycline (64 μg/mL) in AB145. The known efflux pump inhibitor CCCP (10 × 10^−5^ M) was used as a positive control. Untreated bacteria acted as the control. (H) Comedication with baicalein (125 μg/mL) suppressed biofilm formation in AB145. Three biologically independent experiments were conducted, and the data are means ± SD. Nonparametric one-way ANOVA was performed to determine the *P* values (NS, not significant; *, *P < *0.05; **, *P < *0.01; ***, *P < *0.001).

According to the transcriptomic results, the NADH genes necessary for the TCA cycle were downregulated after combination treatment compared to after treatment with doxycycline alone. In agreement with this result, the supplementation of baicalein decreased the NAD^+^/NADH ratio, which indicated that the TCA cycle activity was enhanced when combination treatment was administered (Fig. 7A; Fig. S4A and S5A). Enhanced TCA cycle activity in bacteria is usually accompanied by ROS generation (25). It is immediately followed by increased intracellular ROS levels and reduced superoxide dismutase (SOD) levels in combined treatment (Fig. 7B; Fig. S4B and S5). These results indicated that baicalein combined with doxycycline can increase intracellular oxidative damage by promoting the production of intracellular ROS in bacteria, eventually causing their death.

The bacterial outer membrane is fluid, which is critical for their proliferation and survival (26). We found that the combination treatment resulted in a sharp decrease in cell membrane fluidity, and this process disrupted bacterial intracellular homeostasis, including PMF dissipation (ΔpH) and membrane depolarization (ΔΨ) (Fig. 7C to E; Fig. S4C to E and S6A to C). In addition, the antimicrobial activity of baicalein decreased as the pH (5.5 to 9.0) of Mueller-Hinton broth (MHB) increased, because intracellular ΔpH responds to changes in extracellular pH (Fig. S6D). The uptake of aminoglycoside antibiotics depends on ΔΨ (27). The MIC of gentamicin decreased with an increase in the concentration of baicalein (Fig. S6E). The depolarization of bacterial cell membranes further decreased the production of intracellular ATP (Fig. 7F; Fig. S4F and S6F). As membrane efflux pumps require energy from ATP, in the combination treatment, ethidium bromide (EtBr) efflux and biofilm formation were lower (Fig. 7G and H; Fig. S4G and H and S6G). These findings indicated that baicalein combined with doxycycline can inhibit bacterial multidrug efflux and biofilm formation.

### Baicalein mitigates LPS-induced inflammatory response *in vitro*.

**FIG 8:**
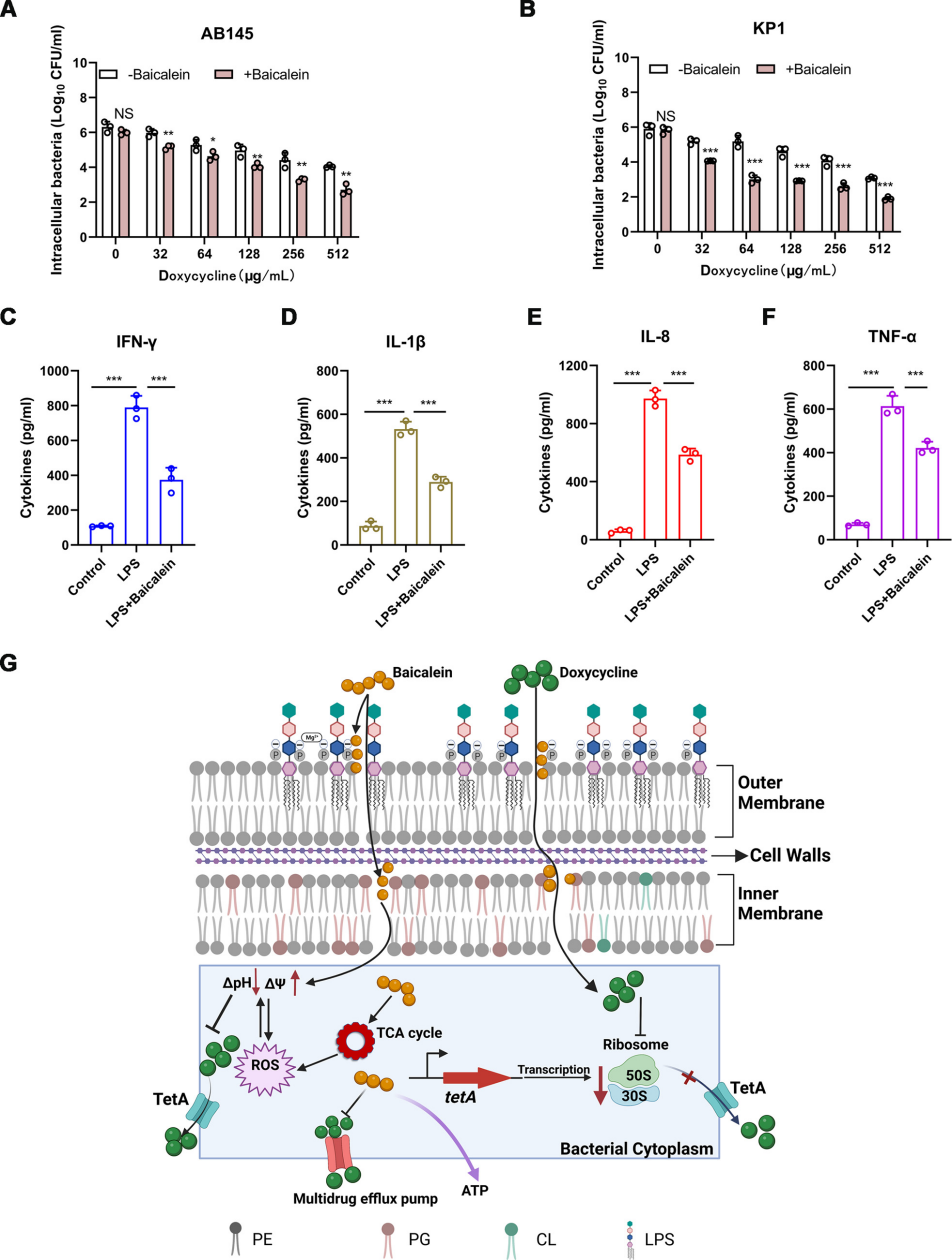
Baicalein attenuated the inflammatory response induced by LPS. (A and B) The combinatorial treatment of baicalein (125 μg/mL) and doxycycline (0 to 512 μg/mL) for 3 h reduced the intracellular bacterial load of A. baumannii AB145 or K. pneumoniae KP1 in 16HBE cells compared to doxycycline treatment alone. (C to F) Acute inflammation triggered by bacterial LPS (1 μg/mL) was alleviated by baicalein (125 μg/mL). After pretreatment with baicalein (125 μg/mL) for 30 min, Raw264.7 cells were stimulated with LPS for 24 h. ELISA was performed to estimate the cytokine levels in culture samples after incubation. Three biologically independent experiments were conducted, and the data are means ± SD. The *P* values were evaluated by conducting unpaired *t* tests between groups or one-way ANOVA among multiple groups (NS, not significant; *, *P < *0.05; **, *P < *0.01; ***, *P < *0.001). (G) Mechanism of action of baicalein combined with doxycycline against *tetA*-positive Gram-negative bacteria. Baicalein can restore the susceptibility to doxycycline of MDR Gram-negative bacteria via membrane-mediated processes by interacting with LPS in the outer membrane and targeting phospholipids in the cytoplasmic membrane. These processes cause membrane depolarization and malfunction, which in turn leads to metabolic alterations such as an increase in the TCA cycle activity and oxidative damage. Doxycycline can also bind to bacterial ribosomes and inhibit the production of bacterial proteins such as TetA.

Nonobligately intracellular bacteria, such as A. baumannii and K. pneumoniae, might avoid host immune defense clearance and antibiotic killing by invading and surviving in host cells (28, 29). Baicalein (125 μg/mL) in combination with doxycycline (0 to 512 μg/mL) decreased the intracellular bacterial load in 16HBE cells more effectively than doxycycline alone (Fig. 8A and B). Then, we used LPS to evoke an inflammatory reaction that simulates a bacterial infection in Raw264.7 cells. The exogenous addition of baicalein strongly suppressed the generation of LPS-mediated proinflammatory factors *in vitro* (Fig. 8C to F). Thus, baicalein treatment can inhibit bacterial invasion of host cells and modulate the host immune system to enhance immunity against certain infections.

### Baicalein improves doxycycline efficacy *in vivo*.

**FIG 9:**
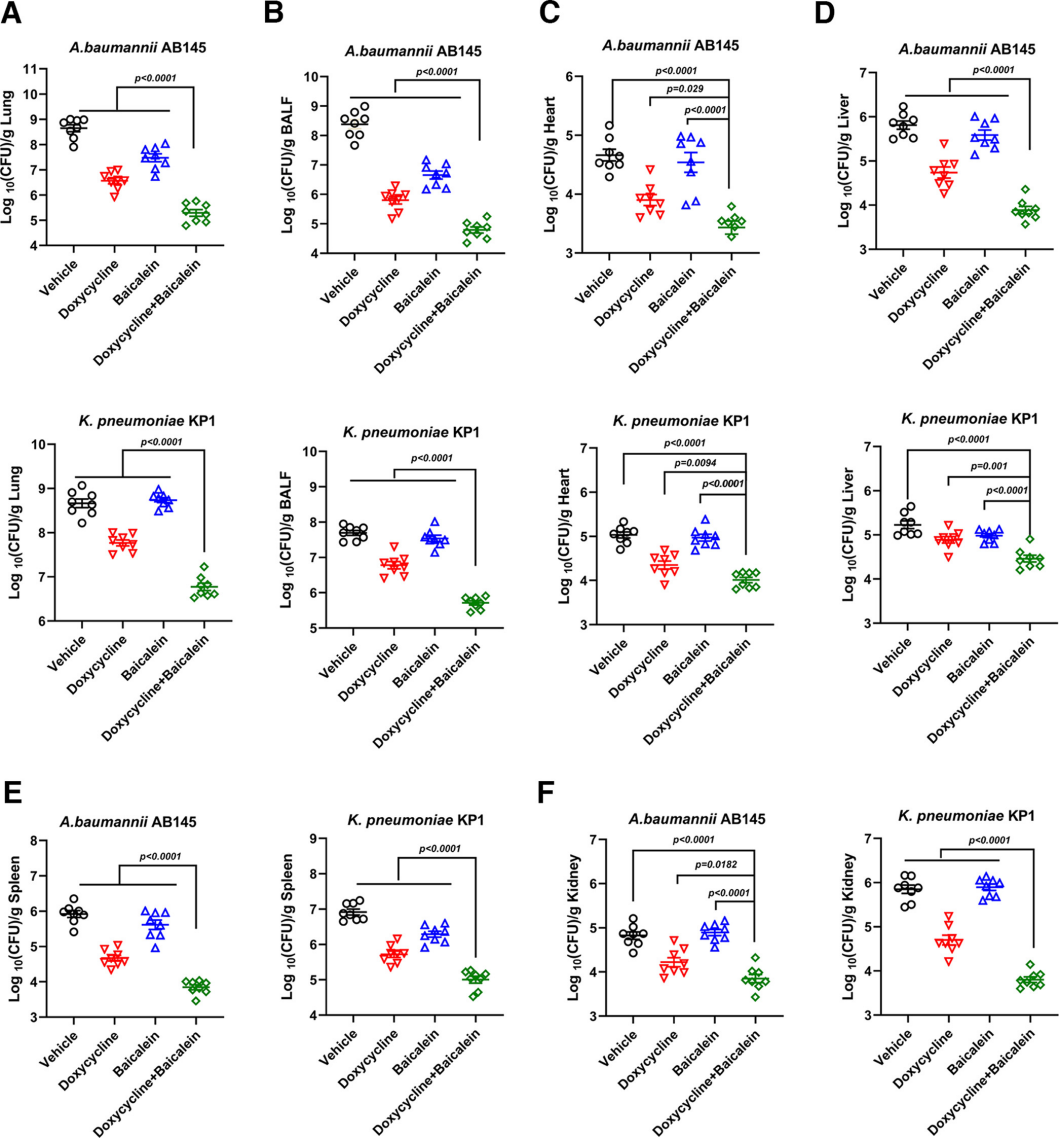
Combinatorial therapy decreased bacterial load in the neutropenic mouse lung infection model. Neutropenic female BALB/c mice (8 per group) were administered a nonlethal dose of Gram-negative bacteria (1.0 × 10^6^ CFU) to the lungs, and 2 h later, the mice were treated intraperitoneally with a single dose of baicalein (50 mg/kg), doxycycline (50 mg/kg), or the combination or with PBS; all mice were euthanized by cervical dislocation after 48 h. In the lung (A), bronchoalveolar lavage fluid (BALF) (B), heart (C), liver (D), spleen (E), and kidney (F), bacterial burdens (log_10_ CFU of AB145 or KP1) were detected. Three biologically independent experiments were conducted, and data are means ± SD. The *P* values were determined by performing two-sided Mann-Whitney U tests.

Baicalein combined with doxycycline showed an effective antibacterial activity *in vitro* (Fig. 8G), which prompted further analysis of this combinatorial treatment in infection models. We used a neutropenic mouse lung infection model to evaluate the effects of this combinatorial treatment *in vivo*. Following the addition of baicalein, the bacterial loads in the bronchoalveolar lavage fluid and mouse organs were the lowest in the combination treatment group (Fig. 9). Additionally, the levels of four inflammatory cytokines (interleukin 1β [IL-1β], gamma interferon [IFN-γ], tumor necrosis factor alpha [TNF-α], and IL-8) in serum were considerably lower after 48 h of pulmonary infection in the combination treatment group than in other groups, which was confirmed by the alleviated pulmonary pathology (Fig. S7). These results indicated that baicalein combined with doxycycline can inhibit bacterial invasion into host cells and reduce the host inflammatory response to these infections *in vivo*.

## DISCUSSION

Baicalein is a bioactive flavonoid extracted from the roots of *S. baicalensis* Georgi. Previous studies have reported that baicalein not only exhibits synergistic effects with antibiotics (15–17) but also has anti-inflammatory effects (30). However, the detailed mechanism of baicalein combined with antibiotics has a therapeutic effect on bacterial infections, which is not fully understood. In this study, we focused on the bacterial cell membrane, metabolism, efflux pumps, and biofilm formation in two *tetA*-positive MDR Gram-negative clinical isolates, including A. baumannii AB145 and K. pneumoniae KP1, to determine the mechanism by which baicalin resensitizes MDR Gram-negative bacteria to doxycycline. Baicalein bound to phospholipids and inhibited Mg^2+^ from attaching to lipid A to destroy the cell membrane of Gram-negative bacteria. Baicalein also altered bacterial metabolism by enhancing the TCA cycle activity, triggering ROS production, and inhibiting multidrug efflux pumps and biofilm formation. Additionally, baicalein prevented the entry of bacteria into the host cell and attenuated host inflammatory responses to bacterial invasion.

Baicalein exhibited synergistic antibacterial activity by disrupting the inner and outer membranes of Gram-negative bacteria. We found that baicalein inhibited Mg^2+^ bound to lipid A to destroy the outer membrane and targeted phospholipids in the cytoplasmic membrane of the Gram-negative bacteria. Lipid A is a lipid LPS component that can electrostatically bind to divalent cations and provide stability to the outer membrane, which prevents the entry of numerous efficient antibacterial drugs (31). Several studies have suggested that phospholipids are essential targets for antibiotic therapy in Gram-negative bacteria (32, 33). However, the cell membranes of Gram-negative bacteria contain only a small amount of cardiolipin, while phosphatidylethanolamine is abundant in mammalian cells. This limits their utility as therapeutic targets for bacterial infections. Phosphatidylglycerol is the main component of the phospholipid layer of the plasma membrane, but its content in mammalian cell membranes is low (34). Thus, phosphatidylglycerol might be an ideal antibacterial target. The effectiveness and low toxicity of baicalein confirmed this hypothesis.

Baicalein altered bacterial metabolism and decreased multidrug efflux pump activity to potentiate the antibacterial activity of doxycycline. The activity of the TCA cycle and ROS production increased in the presence of baicalein in A. baumannii and K. pneumoniae. The production of ROS is a common mechanism by which bactericidal antibiotics induce cell death (35). Meanwhile, bacteria have evolved efflux pump systems to defend themselves against antibiotics. After baicalein treatment, the PMF and ATP levels, which provide the energy required for the drug efflux pump, were reduced. Additionally, disturbances in the intracellular environment caused by damage to the bacterial cell membrane can lead to further PMF depletion (36). The inactivation of efflux pumps can disrupt biofilm formation and pathogenicity in Gram-negative bacteria (37, 38). Baicalein alone or combined with vancomycin has an anti-quorum sensing effect (30, 39). Quorum sensing plays an essential role in biofilm formation, consistent with our results that biofilm formation decreases in the presence of baicalein. Our findings suggested that the above-mentioned mechanisms enhanced doxycycline accumulation in bacterial cells and caused the death of bacteria.

Baicalein combined with doxycycline significantly increased the elimination of *tetA*-positive Gram-negative bacteria *in vivo*. This study showed that the combined treatment mitigated LPS-elicited endotoxin-mediated inflammatory responses. A study showed that baicalein can suppress ROS generation and JAK/STAT activation in macrophages, thus decreasing LPS-mediated inflammation (40). Baicalein can also decrease the activation of the NF-κB and MAPK pathways to mitigate the inflammatory response in macrophages infected with P. aeruginosa (30). However, further studies need to be performed to elucidate the immunomodulatory mechanism by which baicalein resists bacterial infections.

In conclusion, our findings are the first to show that baicalein sensitizes *tetA*-positive MDR Gram-negative pathogens to doxycycline by direct engagement with phospholipids and lipopolysaccharides, thus disturbing the permeability of the inner and outer cell membranes of Gram-negative bacteria. Baicalein addition perturbed the intrinsic antibiotic tolerance mechanisms of Gram-negative bacteria and reduced the host inflammation response triggered by these bacterial infections. Nevertheless, some limitations of this study should be addressed in the future. We found that baicalein can significantly reduce the MIC of doxycycline for Gram-negative isolates; however, an excessive concentration of baicalein was used in the combination, which makes it challenging to reach effective drug concentrations in the plasma of patients. Future research should focus on optimizing the pharmacologic properties of baicalein to enhance its antimicrobial activity and bioavailability as an independent antibiotic or potentiator.

## MATERIALS AND METHODS

### Bacteria, cells, and chemicals.

All strains used in this study are presented in Table 2. Based on previous studies, *tetA* and *tetB* were determined by multiplex PCR in clinical strains (41, 42). Acinetobacter baumannii AB43Δ*crispr-cas* (XDR) and the quality control strain A. baumannii ATCC 19606 were maintained in our laboratory (20). All strains were cultivated in MHB (Oxoid Ltd., Cambridge, UK) at 37°C and 220 rpm unless stated otherwise. The 16HBE and THP-1 cells were maintained in RPMI 1640 medium (Gibco) supplemented with 10% heat-inactivated fetal bovine serum (FBS) (Invitrogen) and 50 μM β-mercaptoethanol. RAW264.7 and A549 cells were cultured in Dulbecco’s modified Eagle medium (DMEM) (Gibco) with 10% heat-inactivated FBS (Invitrogen). Baicalein and antibiotics were obtained from Selleckchem (Shanghai, China). According to methods published by the Clinical and Laboratory Standards Institute (CLSI, USA), baicalein was dissolved in dimethyl sulfoxide (DMSO) with a final concentration of less than 1%.

### Antibacterial susceptibility and synergy testing.

Serial 2-fold dilutions of each antimicrobial agent were performed to determine microdilution MICs in the broth, following the CLSI 2021 guidelines (43). Tested antibiotics were added to triplicate wells of 96-well flat-bottomed tissue culture plates (Corning), and 2-fold serial dilution was performed, followed by the addition of the prepared bacterial inoculum (0.5 × 10^6^ CFU/mL). After incubation for 18 h at 37°C, the minimum concentration of antibiotic without visible bacterial growth was recorded as the MIC. HCl or NaOH was added to the medium to adjust the pH to 5 to 9.

Checkerboard assays were performed on a 6-by-10 dose-response combination matrix to determine the synergy *in vitro*. FICI was calculated as the MIC of the combination divided by the MIC of each compound separately (44). The calculated FICIs were defined as synergistic (FICI ≤ 0.5), additive (0.5 < FICI < 1), indifferent (1 ≤ FICI < 4.0), or antagonistic (FICI ≥ 4.0).

### Bacterial growth inhibition assay.

An overnight culture was diluted with sterile MHB at 1:100 and incubated for 4 h (exponential phase) at 37°C with shaking at 220 rpm. Then, sub-MICs of baicalein (125 μg/mL) and doxycycline (64 μg/mL) were added to the bacterial suspension separately or in combination and incubated for 24 h. At the corresponding time, differently treated cocultured aliquots of the bacterial culture were collected, serially 10-fold diluted, and then spotted onto Mueller-Hinton agar medium (MHA; Oxoid Ltd., Cambridge, UK) plates for calculating the number of CFU. The viability of the bacteria was determined by the number of CFU per milliliter after incubation for 16 h (45).

### Bacterial live/dead staining.

Bacteria cultured in the logarithmic phase of growth were diluted to 1 × 10^6^ CFU/mL in MHB. The bacterial suspensions were incubated with baicalein (62.5 μg/mL) or doxycycline (32 μg/mL) alone or in combination for 8 h. The cells were then harvested by centrifugation and resuspended in sterile phosphate-buffered saline (PBS). LIVE/DEAD BacLight bacterial viability kits (Molecular Probes) containing SYTO9 and PI were used to distinguish between live and dead bacteria. The samples were visualized using a Zeiss Axio Scope A1 upright microscope, and the images were processed for merging using the ImageJ software (46).

### Safety evaluation.

Defibrinated sheep blood cells (8%) were coincubated with different concentrations of combinations for 1 h. After incubation, the optical density at 576 nm (OD_576_) was used to determine hemolytic activity, and the hemolytic rate (as a percentage) was calculated as follows: [(OD_sample_ − OD_blank_)/(OD_0.2%TritonX-100_ − OD_blank_)] × 100 (47). The cytotoxic effects on A549, RAW264.7, and THP-1 cells were assessed by performing the Cell Counting Kit-8 (CCK-8) assay following specific protocols (Beyotime, Shanghai, China). When the cells reached 80% to 90% confluence, the medium was discarded, and the cells were washed with PBS three times and cultured in the corresponding serum-free medium (48–50). Doxycycline (0 to 128 μg/mL) in combination with baicalein (2 to 250 μg/mL) was added to 96-well plates along with 1 × 10^4^ cells, which were cultured for 24 h at 37°C. After 24 h of culture, the CCK-8 solution (10 μL/well) was added to each well. The OD values were determined at 450 nm to evaluate the cytotoxic effect of doxycycline combined with baicalein.

### Resistance development studies.

Bacterial cultures (0.5 × 10^6^ CFU/mL) containing doxycycline (0.25× MIC) alone or in combination with baicalein (62.5 μg/mL) were incubated at 220 rpm for 24 h (37°C). Subsequently, the strains with visible growth (OD_600_ ≥ 0.3) were passaged on antibiotic-free MHA plates for 24 h, and the corresponding MICs of doxycycline were detected by the 2-fold serial dilution method described above. The bacterial suspension (OD_600_ = 0.5) was diluted 1:100 with MHB and incubated in the presence of doxycycline (1/4 MIC) alone or in combination with baicalein (62.5 μg/mL) for the next generation. This step was performed repeatedly for 30 days (51). Sangon Biotech sequenced all mutant strains. All sequence alignment and single-nucleotide polymorphism (SNP) identification were carried out with MEGA 5.0 software (52).

### Cell inner and outer membrane integrity assay.

Fresh bacterial cultures (OD_600_ = 0.5) were incubated with the indicated concentrations of baicalein or polymyxin B (PB; 16 μg/mL) at room temperature for 30 min. Next, the bacterial suspensions were mixed with 10 nmol/L of PI (Solarbio, Beijing, China) to detect the permeabilization of the inner membrane, and then the suspensions were coincubated for 30 min in the dark. The fluorescence was detected at emission/excitation wavelengths of 615/535 nm (46). In the fluorescence or luminescence assay, the corresponding concentrations of drugs (without cells) were incubated with probes as blank controls, and the relative fluorescence intensity was calculated as the value of the analyte minus the value of the blank control.

For the outer membrane permeability assay, 10 μmol/L of NPN (Solarbio, Beijing, China) was used and the assay was performed following the same procedure, with colistin (4 μg/mL) used as a positive control. The fluorescence intensity was measured at emission/excitation wavelengths of 420/350 nm (44).

### Extracellular β-galactosidase measurement.

Different concentrations of baicalein or PB (16 μg/mL) were coincubated with bacterial suspension (OD_600_ = 0.5) for 30 min (37°C). Then, 190 μL of supernatants (12,000 × *g*, 15 min, 4°C) was incubated with 2-nitrophenyl-β-d-galactopyranoside (ONPG; 3 mmol/L; Sigma) for 30 min, and OD_420_ was detected (53).

### Measurement of ATP content.

The bacterial suspension (OD_600_ = 0.5) was coincubated with the indicated concentrations of drugs for 30 min. Then, the lysed bacterial precipitates and bacterial supernatants (12,000 × *g*, 15 min, 4°C) were used to determine the intracellular and extracellular ATP content, respectively (Beyotime; catalog no. S0027).

### SEM.

Doxycycline (64 μg/mL), baicalein (125 μg/mL), or their combination was used to treat bacterial suspension (OD_600_ = 0.5) for 1 h, followed by overnight incubation with 2.5% glutaraldehyde at 4°C. After thorough rinsing three times using PBS, the bacteria were subjected to gradual dehydration in the sequence of 30%, 50%, 70%, 80%, 90%, and 95% ethanol for 10 min each and 100% ethanol for 10 min three times. Subsequently, the samples were dried in a critical point dryer, coated with gold-palladium using an ion sprayer (54), and then observed with SEM (GeminiSEM 300).

### LPS, cationic-ion, and phospholipid assays.

Bacterial LPS was extracted using a kit from iNtRON Biotech (Gyeonggi-Do, South Korea). Next, LPS, baicalein, doxycycline, and bacterial suspensions (5 × 10^8^ CFU/mL) were added to a 96-well plate, followed by 18 h of incubation at 37°C. The checkerboard microdilution assay was performed to assess how LPS (0 to 128 μg/mL) affected the antibacterial activity of baicalein. MIC assays were performed to determine the impact of different cationic ions (50 μg/mL), including KCl, CaCl_2_, MgCl_2_, CuSO_4_, FeCl_3_, NaCl, MnSO_4_, and ZnSO_4_ (Aladdin, Shanghai, China), on the antibacterial activity of baicalein on A. baumannii or K. pneumoniae. PG (Sigma-Aldrich; 841188P; ≥99%), PC (Sigma-Aldrich; catalog no. 840051P; ≥99%), CL (Sigma-Aldrich; 841199P; ≥99%), and PE (Sigma-Aldrich; 840027P; ≥99%) were dissolved in methanol. The checkerboard microdilution assay was performed to determine the effects of different phospholipid concentrations (0 to 128 μg/mL) on the antimicrobial activity of baicalein in MHB.

### RT-PCR and transcriptomic analysis.

A bacterial suspension with an OD_600_ of 0.5 was treated with the indicated concentrations of baicalein at 37°C for 30 min. Total RNA extraction and cDNA reverse transcription were conducted following a method described in another study (20). The mRNA expression of *tetA* (F, 5′-CTTGCCCCTAACCAACCGAACC-3′; R, 5′-AGGCCGTTTGCTTTCAGGGATC-3′) or *mgtA* (F, 5′-ACGTGAAGTGGCAATTGAGG-3′; R, 5′-TACCGTTACACCATGGGCAT-3′) in each sample was detected by 7500 Fast real-time PCR with SYBR green (Applied Biosystems, CA, USA) (44). The 16S rRNA served as an internal reference, and the 2^–ΔΔ^*^CT^* method was applied to determine the relative gene levels.

Acinetobacter baumannii AB145 was grown in MHB to the early exponential phase. Next, bacterial cells were treated with 64 μg/mL doxycycline and 125 μg/mL baicalein independently or in combination for 4 h (55). Total RNA was extracted from these samples, and sequencing was performed using an Illumina HiSeq system (Motif Zhigu Biotechnology, Nanjing, China). DESeq2 was used to normalize raw read counts for estimating gene levels and determining differentially expressed genes (DEGs) with the thresholds of a false discovery rate (FDR) of <0.05 or a fold change (FC) of ≥2 with a *P* value of <0.05. The Cuffdiff software (http://cufflinks.cbcb.umd.edu/) was used to analyze the differences between treatments.

### TCA cycle measurement.

Doxycycline (0 to 512 μg/mL) with or without baicalein (125 μg/mL) was added to bacterial suspensions (OD_600_ = 0.5). The bacterial pellets were washed with PBS after 1 h of incubation, followed by resuspension in 200 μL prechilled extraction buffer. The lysates were centrifuged at 12,000 × *g* for 10 min at 4°C, and the supernatants were analyzed using the NAD^+^/NADH assay kit with water-soluble tetrazolium salt-8 (WST-8, Beyotime, China) (56).

### Total ROS, SOD, and H_2_O_2_ measurement.

The bacterial suspension (OD_600_ = 0.5) was treated with 0.01 mM 2′,7′-dichlorodihydrofluorescein diacetate (DCFH-DA; Invitrogen) to measure ROS levels for 30 min at 37°C. The bacterial suspension (190 μL) mixed with 10 μL of the indicated concentrations of baicalein was added to 96-well plates and incubated for 30 min. The fluorescence intensity was determined at excitation/emission wavelengths of 488/525 nm (57).

The hydrogen peroxide (H_2_O_2_) and SOD activities after treatment with baicalein, doxycycline, or their combination was determined with a hydrogen peroxide assay kit (Beyotime, Shanghai, China) and a total superoxide dismutase assay kit with WST-8 (Beyotime, Shanghai, China).

### Membrane fluidity assay.

Laurdan (10 nmol/L) was added to 5 × 10^8^ CFU/mL of bacterial suspension and incubated at 37°C for 10 min. Next, the bacterial culture was mixed with various concentrations of baicalein or baicalein combined with doxycycline, colistin (4 μg/mL), or benzyl alcohol (50 mmol/L) for 30 min. The generalized polarization (GP) of laurdan was calculated as (*I*_440_ − *I*_490_)/(*I*_440_ + *I*_490_), where *I*_440_ and *I*_490_ represent the emission intensity at 440 and 490 nm, respectively, when excitation was at 350 nm (58).

### PMF and membrane depolarization assay.

Bacterial suspension with an OD_600_ of 0.5 was coincubated with 0.2 × 10^−6^ M of 2’,7’-Bis (2-carboxyethyl-5(6)-carboxyfluorescein) (BCECF)-AM (acetoxymethyl ester) or 0.5 × 10^−3 ^mM of 3,3-dipropylthiadicarbocyanine iodide [DiSC_3_(5); Sigma-Aldrich] for 30 min at 37°C. Then, 10 μL of different drugs, glucose (2.5 × 10^−5^ M), or melittin (16 μg/mL) was added as a supplement and incubated for 30 min, and the fluorescence values of PMF and dissipated bacterial ΔΨ were determined by excitation/emission at 500/522 nm and 622/670 nm, respectively (46).

### EtBr efflux and biofilm formation analysis.

The bacterial suspension (OD_600_ = 0.5), EtBr (5 × 10^−6^ M), drugs at specific concentrations, or the efflux pump inhibitor carbonyl cyanide *m*-chlorophenylhydrazone (CCCP; 10 × 10^−5^ M) was incubated at room temperature. Then, efflux pump activity was monitored by taking readings at intervals of 5 min for 1 h at emission/excitation wavelengths of 600/530 nm.

Bacterial suspensions (5 × 10^5^ CFU/mL) containing equivalent sub-MICs of different drugs were cultivated in triplicate on sterile flat-bottomed 96-well plates and incubated at 37°C for 24 h. The planktonic bacteria were removed, rinsed, and fixed with 4% paraformaldehyde for 20 min. Then, the wells were washed, air dried, and stained with 0.1% crystal violet for 20 min, followed by a repeated washing/drying process. Finally, 95% ethanol (200 μL) was added to the wells, and the OD_595_ was measured.

### Intracellular bacterial content.

Bacteria (multiplicity of infection [MOI] = 100) were used to infect 16HBE cells, followed by coculturing using doxycycline (0 to 512 μg/mL) with or without a sub-MIC of baicalein at 37°C and 5% CO_2_ for 3 h. Colistin (50 μg/mL) was then added to the cells for 15 min to remove extracellular bacteria. After rinsing twice with PBS, the cells were lysed using RPMI 1640 containing 0.1% Triton X-100, followed by inoculation of serial lysate dilutions in MHA to count CFU.

### Cytokine determination.

The levels of cytokines (TNF-α, IL-8, IL-1β, and IFN-γ) were determined using commercial enzyme-linked immunosorbent assay (ELISA) kits (Beyotime, Shanghai, China) following specific protocols.

### Neutropenic mouse lung infection model.

Female BALB/c mice (6 to 8 weeks old; Comparative Medicine Centre of Yangzhou University, Jiangsu, China) were acclimatized for a week. Cyclophosphamide (150 and 100 mg/kg) was administered to 32 mice (*n* = 8/group) on days 4 and 1 before infection for neutropenic induction. Next, the lungs of the mice were injected with 100 μL of bacterial suspension (1.0 × 10^6^ CFU/mouse) (59). After 2 h of infection, the mice were administered an intraperitoneal injection of PBS, doxycycline (50 mg/kg), baicalein (50 mg/kg), or the combination (50 + 50 mg/kg). After 48 h, each mouse was euthanized by cervical dislocation. Peripheral blood serum samples of mice were collected and stored at −80°C for cytokine detection. The organs of the mice were dissected under sterile conditions, followed by homogenization, serial dilution, and inoculation in MHA for counting CFU. The left upper lung tissues of these mice were excised aseptically for hematoxylin-and-eosin (HE) staining. The mice were maintained according to the guidelines of the Administration of Affairs Concerning Experimental Animals issued by the State Council of the People's Republic of China (14 November 1988). The animal experiments were conducted following the instructions provided by the Guide for the Care and Use of Laboratory Animals (production permit number SCYK2022-0009; use permit number SYXK-2022-0044).

### Statistical analysis.

GraphPad Prism 8 was used for performing statistical analysis. The results were expressed as means and standard deviations (SD). Except when stated otherwise, unpaired *t* tests and one-way analysis of variance (ANOVA) were performed to compare the data from two groups and more than two groups, respectively.

### Data availability.

Our transcriptome sequencing data were deposited in the NCBI database under the accession number PRJNA815471.

## Supplementary Material

Reviewer comments
