# Supplementary material for: Baicalein Resensitizes Multidrug-Resistant Gram-Negative Pathogens to Doxycycline
Source: Microbiol Spectr. 2023 Apr 18;11(3):e04702-22. doi: 10.1128/spectrum.04702-22 (PMC10269726; doi:10.1128/spectrum.04702-22)
Supplement: Supplemental file 1 — Supplemental material. Download spectrum.04702-22-s0001.pdf, PDF file, 1.6 MB [file spectrum.04702-22-s0001.pdf]

Supplementary Table 1 Synergistic activity of baicalein in combination with different classes of antibiotics against AB43

Antibiotic	MIC[a] (μg/ml)	FIC[b] index	MIC[c] with Baicalein (μg/ml)	Potentiation[d] (fold)
Ampicillin	4	0.3125	1	4
Doxycycline	1	0.375	0.25	4
Erythromycin	2	0.1875	0.25	8
Rifampin	0.625	0.5	0.16	4
Minocycline	0.125	0.125	0.0078	16
Tetracycline	1	0.375	0.25	4
Tigecycline	0.5	0.1875	0.03	16
Imipenem	2	0.75	0.5	4
Gentamicin	1	0.375	0.125	8
Cefoperazone and Sulbactam	1	0.3125	0.25	4
Ceftriaxone Sodium	8	1	4	2
Ciprofloxacin	0.3125	0.141	0.005	64
Oxytetracycline	4	0.3125	1	4
Vancomycin	4	0.375	0.5	8

[a,c]The MICs of antibiotics in the absence or presence of 62.5 μg/ml of baicalein, respectively.

[b]Fractional inhibitory concentration (FIC) indices were calculated based on chequerboard broth microdilution assays. Synergy: FIC index ≤ 0.5; additive: 0.5 < FIC index < 1; indifferent: 1 ≤ FIC index < 4.

[d]Potentiation of antibiotic in the presence of 62.5 μg/ml of baicalein.

Supplementary Table 2 The MIC of antibiotics against the mutant strains (μg/mL)

Strains	AMP	ERY	RIF	MIN	TET	TIG	IMI	GEN	CAS	CS	CIP	OXY	VAN
**AB145**	16384	32	0.5	8	64	32	1024	16384	32	512	32	256	64
**AB145-256**	32768	256	2	128	128	128	2048	16384	32	1024	256	512	64
**AB145-512**	65536	512	8	256	128	256	2048	32768	64	2048	256	2048	128
**AB145-1024**	32768	2048	8	512	128	256	4096	32768	128	4096	128	2048	256
**AB145-2048**	65536	64	8	512	128	128	2048	65536	64	2048	256	2048	256
**AB145-4096**	65536	2048	8	512	256	64	4096	65536	128	4096	128	2048	256
**KP1**	16384	128	8	256	64	256	8192	2048	128	4096	256	2048	512
**KP1-256**	32768	128	8	512	128	256	8192	2048	512	8192	256	2048	512
**KP1-512**	32768	256	16	256	128	256	16384	4096	512	8192	512	4096	1024
**KP1-1024**	65536	256	16	512	256	512	16384	2048	256	8192	256	4096	1024
**KP1-2048**	32768	512	32	1024	128	512	8192	8192	256	4096	512	8192	2048
**KP1-4096**	65536	1024	32	512	256	1024	32768	8192	256	16384	1024	8192	1024
**KP1-8192**	65536	2048	64	1024	512	1024	32768	8192	512	16384	1024	4096	2048

Note: AB145-256: doxycycline-resistant mutant (MIC=256 μg/mL); AB145-512: doxycycline-resistant mutant (MIC=512 μg/mL); AB145-1024: doxycycline-resistant mutant (MIC=1024 μg/mL); AB145-2048: doxycycline-resistant mutant (MIC=2048 μg/mL); AB145-4096: doxycycline-resistant mutant (MIC=4096 μg/mL); KP1-256: doxycycline-resistant mutant (MIC=256 μg/mL); KP1-512: doxycycline-resistant mutant (MIC=512 μg/mL); KP1-1024: doxycycline-resistant mutant (MIC=1024 μg/mL); KP1-2048: doxycycline-resistant mutant (MIC=2048 μg/mL); KP1-4096: doxycycline-resistant mutant (MIC=4096 μg/mL); KP1-8192: doxycycline-resistant mutant (MIC=8192 μg/mL).

AMP: Ampicillin; ERY: Erythromycin; RIF: Rifampin; MIN: Minocycline; TET: Tetracycline; TIG: Tigecycline; IMI: Imipenem; GEN: Gentamicin; CAS:

Cefoperazone and Sulbactam; CS: Ceftriaxone Sodium; CIP: Ciprofloxacin; OXY: Oxytetracycline; VAN: Vancomycin.

Supplementary Table 3 Single Nucleotide Polymorphisms (SNPs) within the *tetA* gene in Mutants

Strains	position within gene	WT[a]	mutants	amino acid changes
AB145-256	27/380	G	A	Gly→Arg
	49/380	G	T	Gly→Val
	104/380	C	G	Phe→Leu
	151/380	G	C	Gly→Ala
	161/380	G	A	Met→Ile
	218/380	T	G	Asn→Lys
	257/380	A	T	Lys→Asn
	282/380	G	C	Glu→Gln
	366/380	C	T	Gln→*(termination codon)
AB145-512	12/380	T	A	Cys→Ser
	368/380	CAA	CA	Gln→all downstream amino acids changed after the base deleted
	371/380	T	G	Leu→Trp
AB145-1024	3/380	A	G	Lys→Leu
	4/380	A	C	Lys→His
	7/380	TCG	T	Ser→all downstream amino acids changed after the bases deleted
	8/380	TCG	T	Ser→all downstream amino acids changed after the bases deleted
	10/380	G	C	Trp→Ser
	368/380	A	ATT	Gln→all downstream amino acids changed after the bases inserted
	369/380	CT	T	Leu→all downstream amino acids changed after the base deleted
AB145-2048	10/380	G	T	Trp→Leu
	368/380	A	T	Gln→His
AB145-4096	10/380	G	T	Trp→Phe
	11/380	G	C	Trp→Phe
	12/380	T	A	Cys→Ser
	16/380	A	G	Asp→Gly
	369/380	C	T	Leu→Phe
KP1-256	13/377	GCC	GC	Ala→all downstream amino acids changed after the base deleted
	5/377	T	A	Ser→Glu
	6/377	C	CC	Ser→all downstream amino acids changed after the base inserted

Supplementary Table 3-Continued

Strains	position within gene	WT[a]	mutants	amino acid changes
KP1-256	7/377	A	GA	Ser→all downstream amino acids changed after the base inserted
	12/377	GCC	GC	Ala→all downstream amino acids changed after the base deleted
	364/377	A	T	Gln→His
	370/377	GT	GGT	Gly→all downstream amino acids changed after the base inserted
KP1-512	12/377	C	T	Ala→Val
	370/377	GT	GGT	Gly→all downstream amino acids changed after the base inserted
KP1-1024	11/377	GG	G	Ala→Asp
	12/377	C	A	Ala→Asp
	367/377	TTT	TT	Phe→all downstream amino acids changed after the base deleted
KP1-2048	12/377	C	A	Ala→Asp
	364/377	A	T	Gln→His
	366/377	T	G	Phe→Trp
	367/377	T	G	Phe→Trp
KP1-4096	13/377	C	G	Ala→Ala
	368/377	G	T	Gly→Leu
	369/377	GT	TGT	Gly→all downstream amino acids changed after the base inserted
KP1-8192	6/377	CA	CGA	Ser→all downstream amino acids changed after the base inserted
	10/377	GTG	GT	Val→all downstream amino acids changed after the base deleted
	12/377	C	A	Ala→Asp

Note: SNPs were all confirmed by Sanger sequencing.

[a]WT: wild type, *A. baumannii* AB145 or *K. pneumoniae* KP1.

AB145-256: doxycycline-resistant mutant (MIC=256 μg/mL); AB145-512: doxycycline-resistant mutant (MIC=512 μg/mL); AB145-1024: doxycycline-resistant mutant (MIC=1024 μg/mL); AB145-2048: doxycycline-resistant mutant (MIC=2048

μg/mL); AB145-4096: doxycycline-resistant mutant (MIC=4096 μg/mL); KP1-256: doxycycline-resistant mutant (MIC=256 μg/mL); KP1-512: doxycycline-resistant mutant (MIC=512 μg/mL); KP1-1024: doxycycline-resistant mutant (MIC=1024 μg/mL); KP1-2048: doxycycline-resistant mutant (MIC=2048 μg/mL); KP1-4096: doxycycline-resistant mutant (MIC=4096 μg/mL); KP1-8192: doxycycline-resistant mutant (MIC=8192 μg/mL).

Supplementary Figures

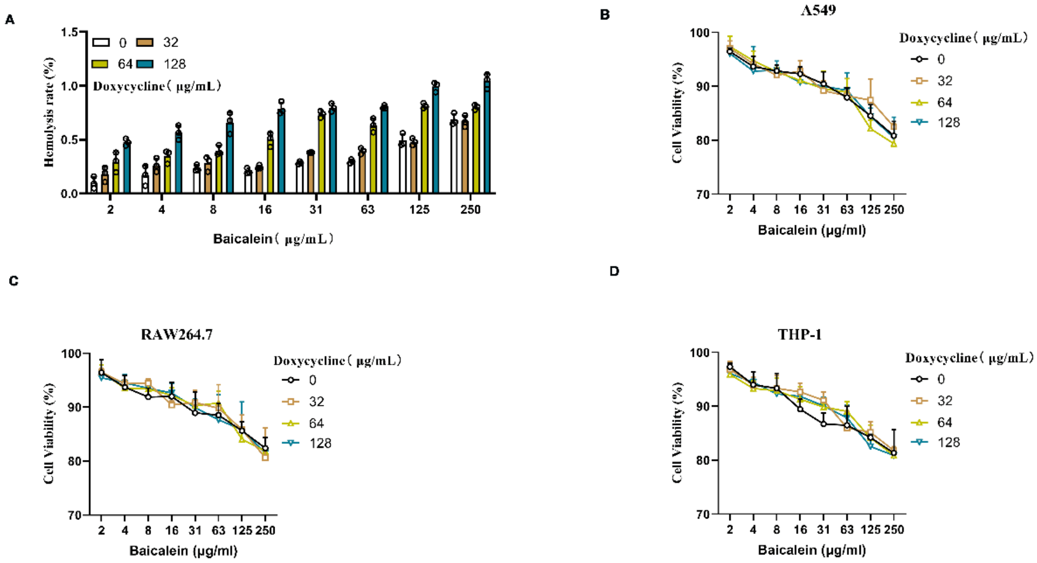

Supplementary Figure 1. **Safety evaluation of baicalein.** Different concentrations of baicalein (2-250 μg/mL) had no significant effects on the hemolytic toxicity of the red blood cells of sheep **(A)** and low cytotoxicity in A549 **(B)**, RAW264.7 **(C)** and THP-1 **(D)** cells caused by doxycycline (0-128 μg/mL). Data are based on three biological replicates and shown to be the mean ± SD.

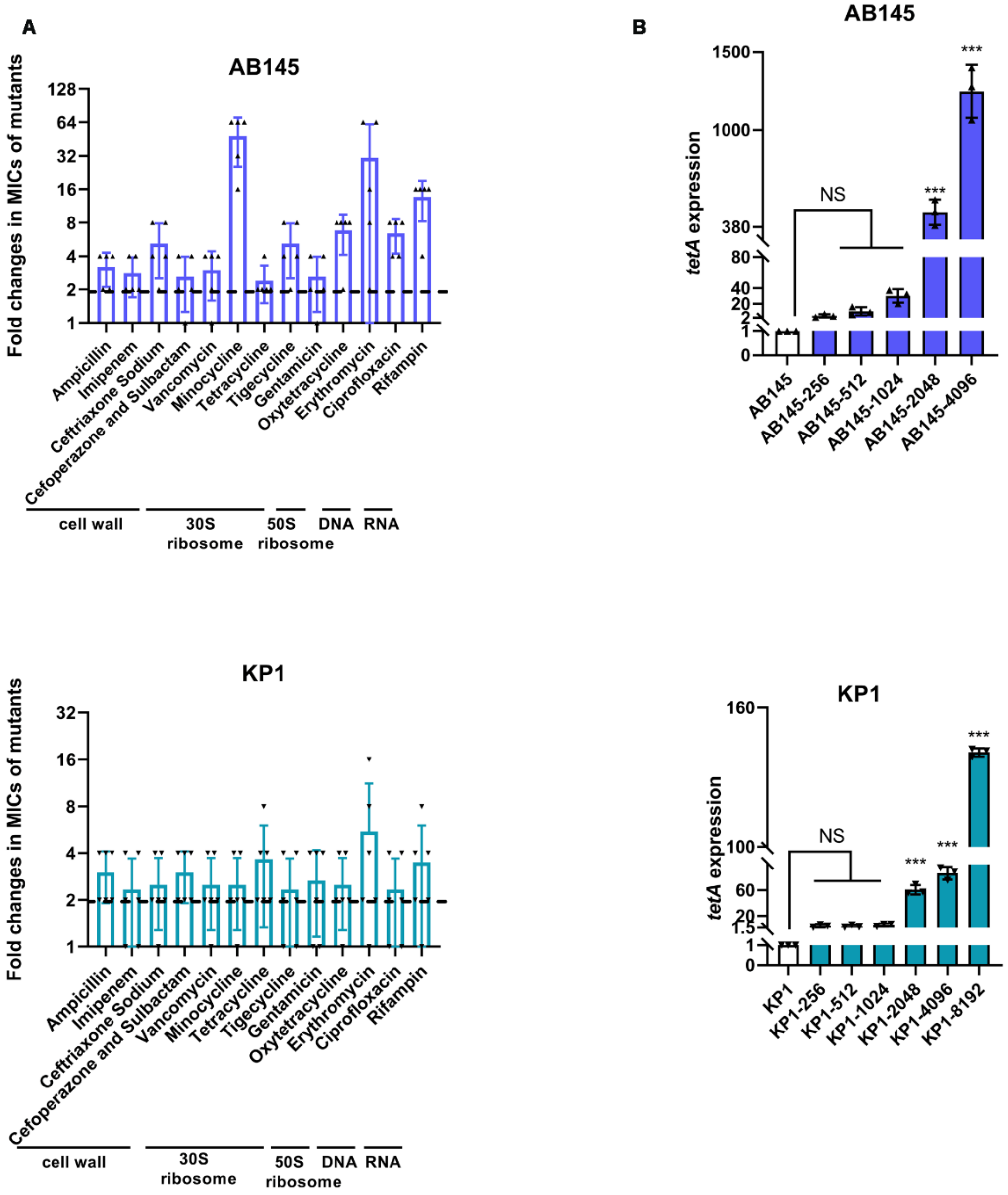

***Supplementary Figure 2.* Doxycycline-resistant mutants showed cross-resistance to other antibiotics**. **(A)** Resistance profile of doxycycline-resistant mutants. A cross-resistance was defined as MICs that were 2-fold higher against mutants than wide-type bacteria. **(B)** The expression of *tetA* mRNA in different doxycycline-resistant mutants. AB145-256: doxycycline-resistant mutant (MIC=256 μg/mL); AB145-512: doxycycline-resistant mutant (MIC=512 μg/mL); AB145-1024: doxycycline-resistant mutant (MIC=1024 μg/mL); AB145-2048: doxycycline-resistant mutant (MIC=2048

μg/mL); AB145-4096: doxycycline-resistant mutant (MIC=4096 μg/mL); KP1-256: doxycycline-resistant mutant (MIC=256 μg/mL); KP1-512: doxycycline-resistant mutant (MIC=512 μg/mL); KP1-1024: doxycycline-resistant mutant (MIC=1024 μg/mL); KP1-2048: doxycycline-resistant mutant (MIC=2048 μg/mL); KP1-4096: doxycycline-resistant mutant (MIC=4096 μg/mL); KP1-8192: doxycycline-resistant mutant (MIC=8192 μg/mL). Data were shown to be the mean ± SD, and differences were assessed using non-parametric one-way ANOVA. (****P* < 0.001; NS, not significant).

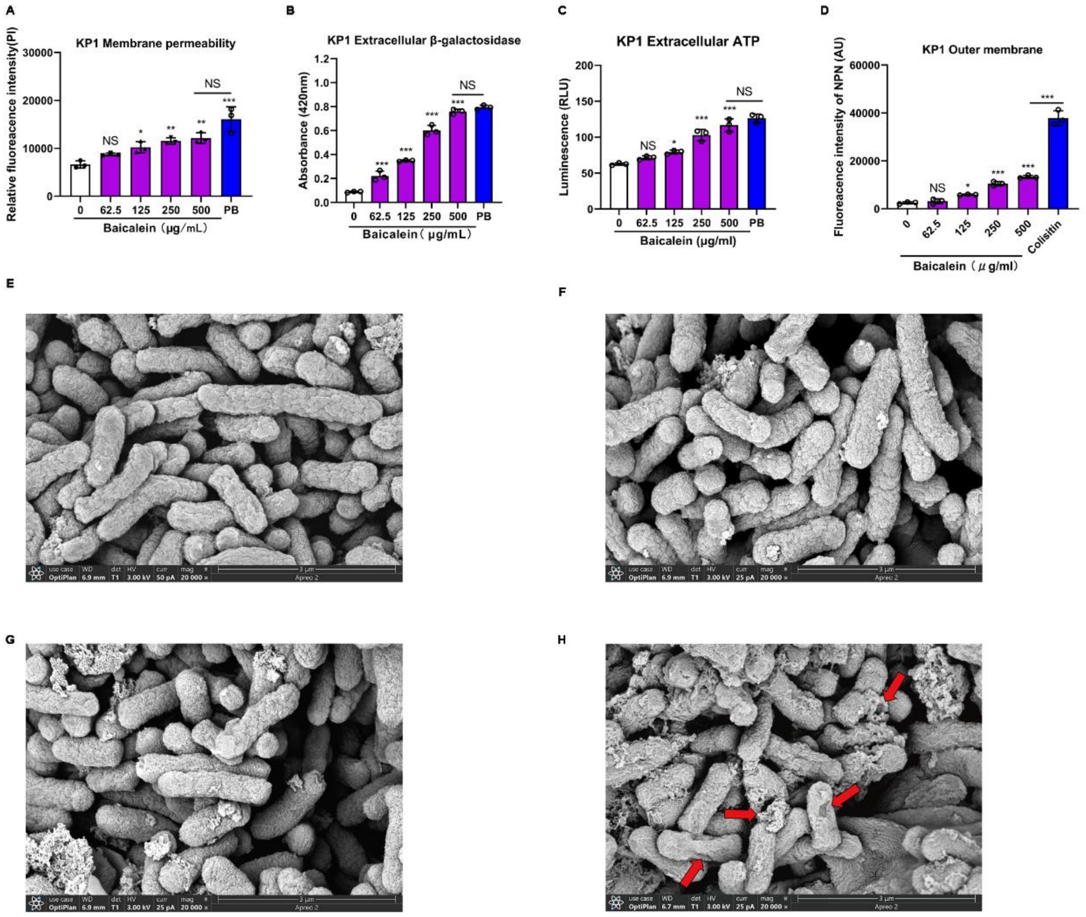

***Supplementary Figure 3*. Baicalein exerts antibacterial activity by targeting the inner and outer membranes of *K. Pneumoniae*. (A)** Increased cytoplasmic membrane permeability of *K. Pneumoniae* KP1 after baicalein treatment (0-500 μg/mL) or PB (polymyxins B, 16 μg/mL) for 30 min, probed with 10 nmol/L of

propidium iodide (PI). **(B and C)** Increased level of bacterial contents, including extracellular **(B)** β-galactosidase (MW = 130 kDa) and **(C)** ATP (MW = 507 Da) in the presence of baicalein (0-500 μg/mL) for 30 min. **(D)** Baicalein disrupts the outer membrane of *K. Pneumoniae* KP1 by measuring the fluorescence intensity of 1-N-phenylnaphthylamine (NPN) after exposure to increasing concentrations of baicalein (0-500 μg/mL) or colistin (4 μg/mL) for 30 min. The data in panels A-D represent three biological replicates, and error bars represent SD. *p*-values were determined using a non-parametric, one-way analysis of variance (ANOVA). **P* < 0.05, ***P* < 0.01, ****P* < 0.001. **(E-H)** Morphological changes of *K. Pneumoniae* KP1 treated with PBS **(E)**; sub-MIC of doxycycline (64 μg/ml) **(F)**; or sub-MIC of baicalein (125 μg/ml) **(G)**; or their combination for 1 h **(H)** visualized with SEM. Scar bar, 3 μm. Red arrows marked the destroyed outer membrane.

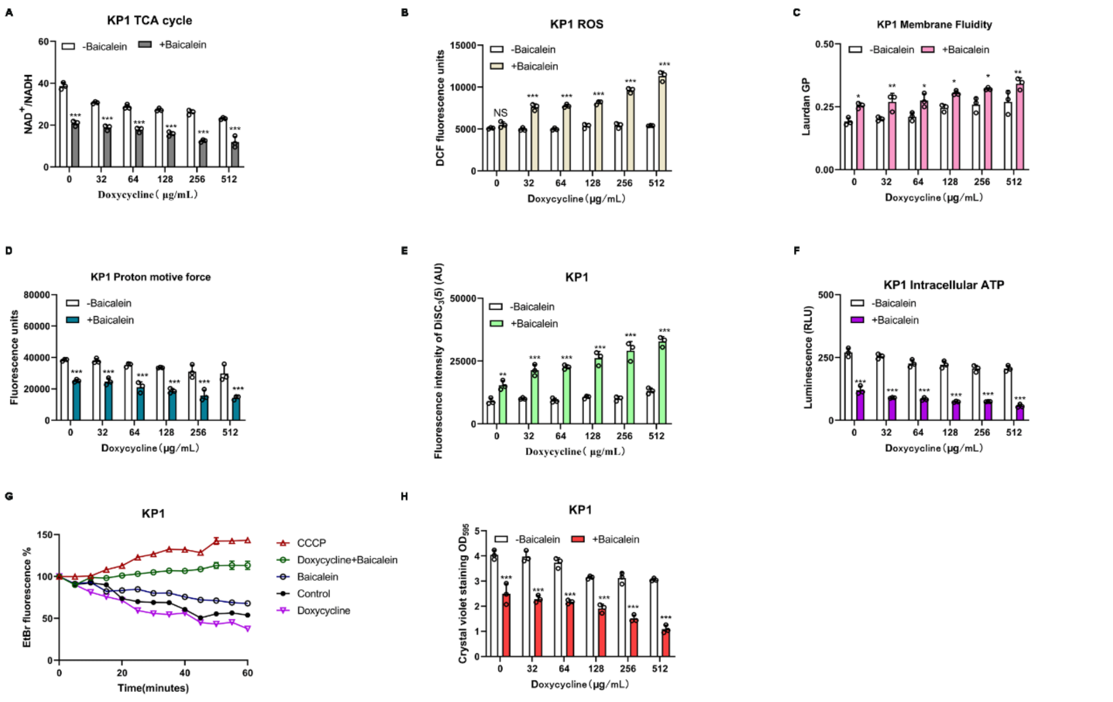

***Supplementary Figure 4.* Mechanism of baicalein combined doxycycline against *K. pneumoniae*.** **(A)** An accelerated TCA cycle was observed under a combination of baicalein (125 μg/mL) and doxycycline (0-512 μg/mL) in *K. pneumoniae* KP1. **(B)**

The administration of the combination accelerates ROS generation in KP1 after treatment for 30 min. **(C)** The membrane fluidity decreased after baicalein (125 μg/ml) colocalization with doxycycline in KP1. **(D)** The combined treatment promotes PMF consumption. **(E)** Baicalein (125 μg/ml) combined with doxycycline promotes KP1 cell membrane potential depolarization. **(F)** Decreased production of intracellular ATP in KP1 treated with combined treatment. **(G)** Baicalein (125 μg/ml) combined with doxycycline (64 μg/ml) effectively inhibited the EtBr efflux pump in KP1. The known efflux pump inhibitor CCCP (10×10^{-5} M) is a positive control. Control represents untreated bacteria. **(H)** Combined treatment suppressed the biofilm formation in KP1. Three biologically independent experiments were conducted, and the mean ± SD is shown. Non-parametric one-way ANOVA was used to determine *P* values (*$P < 0.05$, **$P < 0.01$, ***$P < 0.001$).

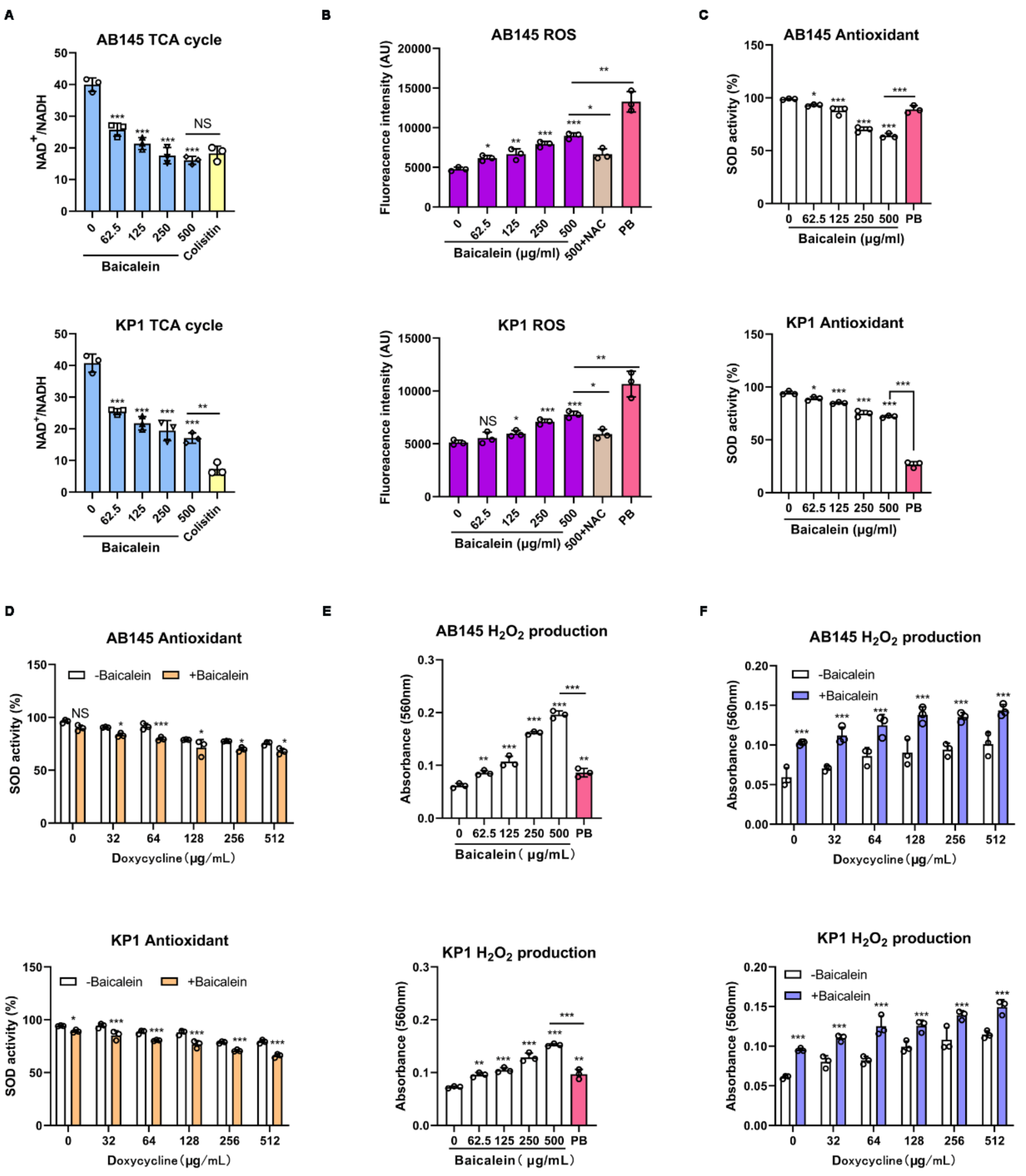

***Supplementary Figure 5.* Baicalein promotes the production of reactive oxygen species in a dose-dependent manner. (A)** Baicalein or colistin (4 μg/mL) enhances TCA cycle activity. **(B)** Accumulated total ROS in *A. baumannii* AB145 and *K. pneumoniae* KP1 treated with baicalein or PB (polymyxins B, 16 μg/mL). Exogenous addition of NAC (N-acetylcysteine, 6 mmol/L) prevented the accumulation of ROS induced by 500 μg/ml of baicalein. **(C and D)** Baicalein reduces the activity of SOD in the absence **(C)** or presence of doxycycline **(D)**. **(E and F)** Baicalein induces the production of H_2O_2 in the absence **(E)** or presence of doxycycline **(F)**. Three

biologically independent experiments were conducted, and the mean ± SD is shown. *P* values were determined by unpaired t-tests between two groups or one-way ANOVAs among multiple groups (*$P < 0.05$, **$P < 0.01$, ***$P < 0.001$).

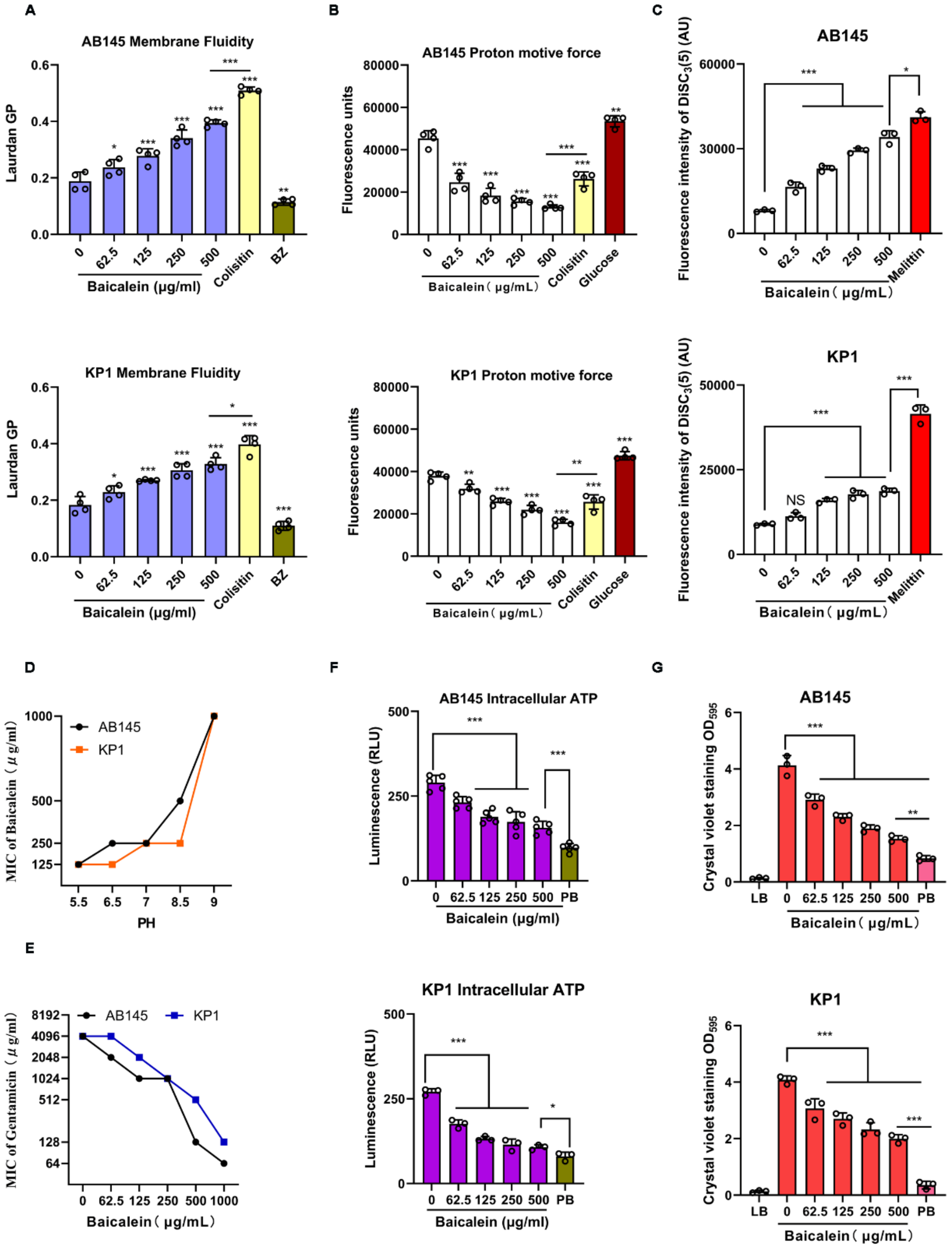

Supplementary Figure 6. **Mechanism of baicalein against Gram-negative bacteria.**

(A) The outer membrane fluidity for *A. baumannii* AB145 or *K. pneumoniae* KP1

after treatment with baicalein or colistin (4 μg/mL) was decreased. 50 mmol/L of benzyl alcohol (BZ) was used as the control. **(B)** Baicalein decreased PMF in a dose-dependent manner. PMF was enhanced by glucose (2.5×10^{-5} M), considered a positive control. **(C)** A dose-dependent dissipation of bacterial membrane potential is observed when $DiSC_3(5)$ fluoresces after 30 minutes of exposure to increasing concentrations of baicalein. Melittin (16 μg/mL) was used as the positive control. **(D)** Baicalein's antibacterial activity is decreased in alkaline media. **(E)** Baicalein decreased the MIC of gentamicin in AB145 or KP1 in a dose-dependent manner. **(F)** Baicalein decreased levels of intracellular ATP in AB145 and KP1. PB (polymyxins B, 16 μg/mL) was used as the positive control. **(G)** Baicalein or PB (polymyxins B, 16 μg/mL) suppressed the biofilm formation in AB145 and KP1. Three biologically independent experiments were conducted, and the mean ± SD is shown. *P* values were determined by unpaired t-tests between two groups or one-way ANOVAs among multiple groups (*$P < 0.05$, **$P < 0.01$, ***$P < 0.001$).

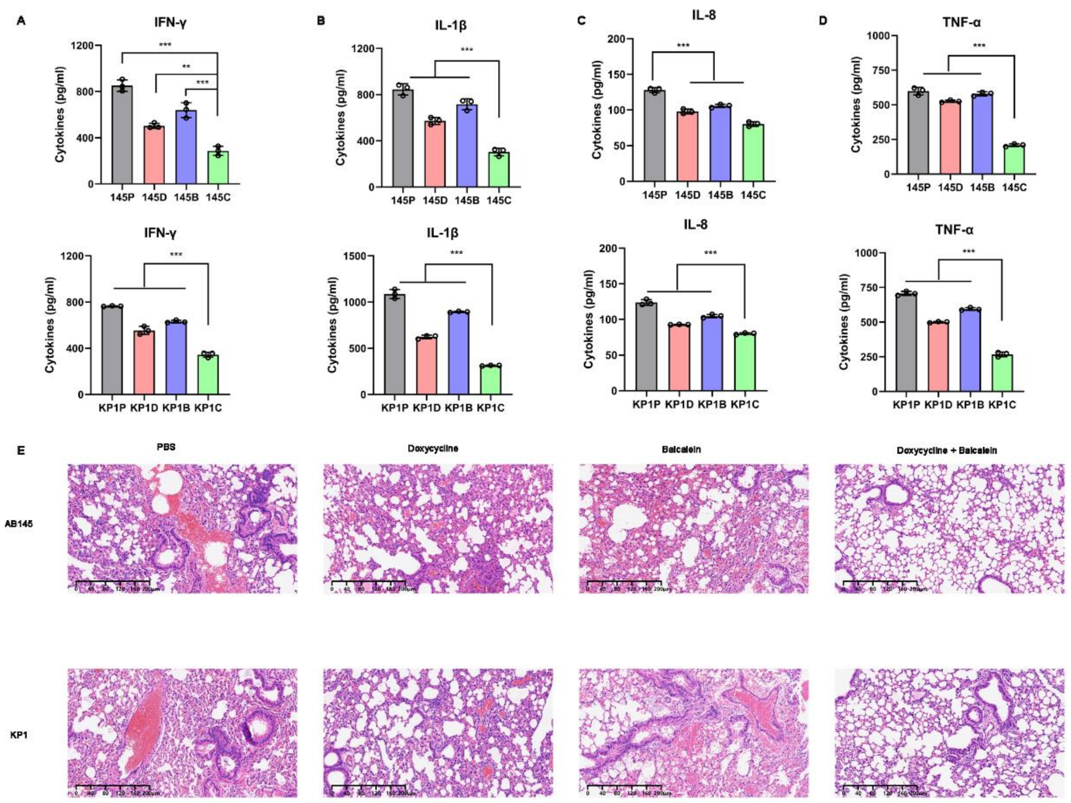

Supplementary Figure 7. **Combining baicalein and doxycycline reduces the inflammatory responses in the neutropenic mouse lung infection model. (A-D)** Baicalein combined with doxycycline reduces AB145 or KP1-induced pro-inflammatory cytokines release in serum. 145P: AB145 + PBS; 145D: AB145 + doxycycline (50 mg/kg); 145B: AB145 + baicalein (50 mg/kg); 145C: AB145 + doxycycline (50 mg/kg) + baicalein (50 mg/kg); KP1P: KP1 + PBS; KP1D: KP1 + doxycycline (50 mg/kg); KP1B: KP1 + baicalein (50 mg/kg); KP1C: KP1 + doxycycline (50 mg/kg) + baicalein (50 mg/kg). Three biologically independent experiments were conducted, and the mean ± SD is presented. One-way ANOVA was used to determine *P* values among multiple groups (**P* < 0.05, ***P* < 0.01, ****P* < 0.001). **(E)** Histopathological changes of lungs in the neutropenic mouse lung infection model and after 2 h post-infection, the mice were treated with PBS, doxycycline (50 mg/kg), baicalein (50 mg/kg), or their combination (50 + 50 mg/kg)

by intraperitoneal injections. Magnification, × 100.
